# Chemoselective Synthesis of Mannich Adducts from 1,4-Naphthoquinones and Profile as Autophagic Inducers in Oral Squamous Cell Carcinoma

**DOI:** 10.3390/molecules28010309

**Published:** 2022-12-30

**Authors:** Amanda A. Borges, Michele P. de Souza, Anna Carolina C. da Fonseca, Guilherme F. Wermelinger, Ruan C. B. Ribeiro, Adriane A. P. Amaral, Cláudio José C. de Carvalho, Lucas S. Abreu, Lucas Nicolau de Queiroz, Elan C. P. de Almeida, Vitor W. Rabelo, Paula A. Abreu, Bruno Pontes, Vitor F. Ferreira, Fernando de C. da Silva, Luana da S. M. Forezi, Bruno K. Robbs

**Affiliations:** 1 Departamento de Química Orgânica, Instituto de Química, Campus do Valonguinho, Universidade Federal Fluminense, Niterói CEP 24020-150, Brazil; 2 Programa de Pós-Graduação em Ciências Aplicadas a Produtos para Saúde, Faculdade de Farmácia, Universidade Federal Fluminense, Niterói CEP 24241-000, Brazil; 3 Programa de Pós-Graduação em Odontologia, Instituto de Saúde de Nova Friburgo, Universidade Federal Fluminense, Nova Friburgo CEP 28625-650, Brazil; 4 Departamento de Ciência Básica, Campus Universitário de Nova Friburgo, Universidade Federal Fluminense, Nova Friburgo CEP 28625-650, Brazil; 5 Instituto de Biodiversidade e Sustentabilidade, Campus Macaé, Universidade Federal do Rio de Janeiro, Macaé CEP 27965-045, Brazil; 6 Instituto de Ciências Biomédicas, Universidade Federal do Rio de Janeiro, Rio de Janeiro CEP 21941-902, Brazil; 7 Departamento de Tecnologia Farmacêutica, Faculdade de Farmácia, Universidade Federal Fluminense, Niterói CEP 24241-000, Brazil

**Keywords:** oral squamous cell carcinoma, lawsone, lapachol, Mannich adducts, pyruvate kinase M2, autophagy

## Abstract

Oral squamous cell carcinoma (OSCC) is a worldwide public health problem, accounting for approximately 90% of all oral cancers, and is the eighth most common cancer in men. Cisplatin and carboplatin are the main chemotherapy drugs used in the clinic. However, in addition to their serious side effects, such as damage to the nervous system and kidneys, there is also drug resistance. Thus, the development of new drugs becomes of great importance. Naphthoquinones have been described with antitumor activity. Some of them are found in nature, but semi synthesis has been used as strategy to find new chemical entities for the treatment of cancer. In the present study, we promote a multiple component reaction (MCR) among lawsone, arylaldehydes, and benzylamine to produce sixteen chemoselectively derivated Mannich adducts of 1,4-naphthoquinones in good yield (up to 97%). The antitumor activities and molecular mechanisms of action of these compounds were investigated in OSCC models and the compound **6a** induced cytotoxicity in three different tumor cell lines (OSCC4, OSCC9, and OSCC25) and was more selective (IS > 2) for tumor cells than the chemotropic drug carboplatin and the controls lapachol and shikonin, which are chemically similar compounds with cytotoxic effects. The **6a** selectively and significantly reduced the amount of cell colony growth, was not hemolytic, and tolerable in mice with no serious side effects at a concentration of 100 mg/kg with a LD_50_ of 150 mg/kg. The new compound is biologically stable with a profile similar to carboplatin. Morphologically, **6a** does not induce cell retraction or membrane blebs, but it does induce intense vesicle formation and late emergence of membrane bubbles. Exploring the mechanism of cell death induction, compound **6a** does not induce ROS formation, and cell viability was not affected by inhibitors of apoptosis (ZVAD) and necroptosis (necrostatin 1). Autophagy followed by a late apoptosis process appears to be the death-inducing pathway of **6a**, as observed by increased viability by the autophagy inhibitor (3-MA) and by the appearance of autophagosomes, later triggering a process of late apoptosis with the presence of caspase 3/7 and DNA fragmentation. Molecular modeling suggests the ability of the compound to bind to topoisomerase I and II and with greater affinity to hPKM2 enzyme than controls, which could explain the mechanism of cell death by autophagy. Finally, the in-silico prediction of drug-relevant properties showed that compound **6a** has a good pharmacokinetic profile when compared to carboplatin and doxorubicin. Among the sixteen naphthoquinones tested, compound **6a** was the most effective and is highly selective and well tolerated in animals. The induction of cell death in OSCC through autophagy followed by late apoptosis possibly via inhibition of the PKM2 enzyme points to a promising potential of **6a** as a new preclinical anticancer candidate.

## 1. Introduction

Oral squamous cell carcinoma (OSCC) accounts for approximately 90% of all oral cancers and is the eighth most common cancer in men [1,2,3]. The main risk factors involved are alcohol, tobacco products, and human papillomavirus infection and it is more common in men over 60 years of age [2,4,5]. An overall five-year survival rate of 65% is estimated for this type of cancer, ranging from 84% for localized cancers to 39% for metastatic cancers [2]. Despite its incidence and mortality, diagnosis usually still occurs in advanced disease [6]. Despite its incidence and mortality, diagnosis usually still occurs in advanced disease [6].

The usual treatments in the clinic for OSCC are radiotherapy, surgery and/or chemotherapy, depending on the stage of the disease [4,5]. According to the American Cancer Society, Cisplatin, Carboplatin, 5-Fluorouracil, Paclitaxel, Docetaxel, and Hydroxyurea are the current chemotherapeutic agents in use. However, serious side effects can occur, including an increased chance of infections, bruising or bleeding, fatigue, and nerve and kidney damage, highlighting the need for new and effective antineoplastic drugs that can improve disease outcome and overcome toxicity.

Naphthoquinones are present in plants, animals and microorganisms and some derivatives have been described with cytotoxic and antitumor activity [7,8]. Their mechanism of action involves mainly DNA damage via the production of reactive oxygen species (ROS), inhibition of topoisomerase II enzyme, reactivation of the p53 suppressor protein, and endoplasmic reticulum stress apoptosis induction [8]. Several research groups are trying to improve the antitumor effects of naphthoquinones by performing chemical modifications. In this context, Mannich’s reactions are widely used in medicinal chemistry since they favor the bioavailability and water solubility of the compounds, and due to their simplicity and scalability [9,10].

In the present study, we promote a multiple component reaction (MCR) among lawsone (**1**), benzylamine (**2**), and arylaldehydes (**3**) to produce sixteen chemoselectively derivated Mannich adducts of 1,4-naphthoquinones in good yield (up to 97%). Moreover, we investigate their antitumor activity and molecular mechanisms in a series of in vitro and in vivo assays using OSCC and normal oral human cell models.

## 2. Experimental Section

### 2.1. Chemistry

#### 2.1.1. Materials and Methods

The reagents were purchased from Sigma-Aldrich Brazil and were used without further purification. Column chromatography was performed with silica gel 60 (Merck 70–230 mesh, Darmstadt, Germany). Analytical thin layer chromatography was performed with silica gel plates (Merck, TLC silica gel 60 F_254_), and the plates were visualized using UV light. The indicated yields refer to homogeneous materials purified by chromatography and confirmed by spectroscopic techniques. Melting points were obtained on a Thermo scientific 9100 apparatus and were uncorrected. Infrared spectra were collected using KBr pellets on a Perkin-Elmer (Waltham, MA, USA) model 1420 FT-IR spectrophotometer, and the spectra were calibrated relative to the 1601.8 cm^−1^ absorbance of polystyrene. ^1^H and ^13^C NMR were recorded at room temperature using a Varian Mercury 300 or Varian Mercury 500 MHz, in the CDCl_3_. The chemical shift data were reported in units of δ (ppm) downfield from solvent, and the solvent was used as an internal standard; coupling constants (*J*) are reported in hertz and refer to apparent peak multiplicities. High-resolution mass spectra (HRMS) were recorded on a MICROMASS Q-TOF mass spectrometer (Waters Corporation, Milford, MA, USA).

#### 2.1.2. General Procedures for Synthesis of Naphthoquinones **5**

A 50 mL round bottom flask containing a solution of **4** (0.6 mmol), potassium carbonate (3.0 mmol) and methylchloroformate (0.66 mmol) in dry THF (15 mL) under argon atmosphere was stirred for 24 h at room temperature. Then, the mixture was filtered under low pressure and washed with dichloromethane. The solution was concentrated in low pressure and the residue was crystallized using a dichloromethane/hexane (or pentane) mixture. The compounds **5a**–**i** were obtained as yellow solids in good yields.

Methyl benzyl((4-chlorophenyl)(3-((methoxycarbonyl)oxy)-1,4-dioxo-1,4-dihydronaphthalen-2-yl)methyl)carbamate (**5a**). Light yellow solid, m.p. 140–142 °C dec., 97% yield. IR (KBr, cm^−1^): ν 2955, 1979, 1774, 1682, 1672, 1448, 1313, 1293, 1269, 1228, 1205, 1188, 1160, 945, 800, 767, 723, 701, 686. ^1^H NMR (500.00 MHz, DMSO-d6) δ ppm: 7.94–7.75 (m, 4H), 7.34 (d, J 8.7 Hz, 2H), 7.28–7.23 (m, 2H), 6.91 (d, J 7.3 Hz, 1H), 6.86 (d, J 1.4 Hz, 1H), 6.82 (d, J 10.5 Hz, 3H), 4.89 (d, J 16.9 Hz, 1H), 4.32 (d, J 16.8 Hz, 1H), 3.68 (s, 3H), 3.68 (s, 3H). ^13^C NMR (125.0 MHz, DMSO-d6) δ ppm: 183.2, 178.2, 157.0, 150.9, 150.8, 138.5, 136.7, 135.4, 134.9, 134.8, 132.5, 131.6, 130.9, 130.2, 129.2, 128.8, 128.6, 128.3, 127.4, 127.2, 127.0, 126.7, 126.5, 126.3, 56.6, 55.0, 53.6, 48.8. HR-ESIMS calc. for C_28_H_22_ClNNaO_7_ [M + Na]^+^: 542.0977. Found: 542.0949. Δ 5.2 ppm.

Methylbenzyl((3-((methoxycarbonyl)oxy)-1,4-dioxo-1,4-dihydronaphthalen-2yl)(phenyl) methyl)carbamate (**5b**). Yellow solid, m.p. 128–130 °C dec., 97% yield. IR (KBr, cm^−1^): ν 2956, 1776, 1673, 1448, 1312, 1295, 1269, 1226, 1189, 1160, 1120, 948, 763, 734, 700, 690, 681. ^1^H NMR (500.00 MHz, DMSO-d6) δ ppm: 7.93–7.88 (m, 2H), 7.87–7.82 (m, 2H), 7.29–7.25 (m, 3H), 7.24–7.21 (m, 2H), 6.94 (t, J 7.7 Hz, 2H), 6.86 (s, 1H), 6.84 (d, J 7.0 Hz, 2H), 4.83 (d, J 16.8 Hz, 1H), 4.40 (d, J 16.8 Hz, 1H), 3.68 (s, 3H), 3.67 (s, 3H); ^13^C NMR (125.0 MHz, DMSO-d6) δ ppm: 183.3, 178.2, 157.0, 150.9, 150.7, 138.8, 137.3, 135.4, 135.3, 134.9, 131.6, 130.2, 128.8, 128.3, 127.7, 127.4, 127.0, 126.6, 126.5, 126.3, 56.6, 55.6, 53.5, 48.8. HR-ESIMS calc. for C_28_H_23_NNaO_7_ [M + Na]^+^: 508.1366. Found: 508.1361. Δ 1.0 ppm.

Methylbenzyl((3-((methoxycarbonyl)oxy)-1,4-dioxo-1,4-dihydronaphthalen-2-yl)(4-nitrophenyl)methyl)carbamate (**5c**). Light yellow solid, m.p. 124–126 °C, 90% yield. IR (KBr, cm^−1^): ν 2952, 1796, 1779, 1688, 1524, 1347, 1284, 1189, 1163, 793, 715, 702, 681. ^1^H NMR (500.00 MHz, DMSO-d6) δ ppm: 8.15 (d, J 8.8 Hz, 2H), 7.91–7.88 (m, 1H), 7.87–7.77 (m, 3H), 7.56 (d, J 8.5 Hz, 2H), 6.94–6.88 (m, 4H), 6.79 (t, J 6.7 Hz, 1H), 5.05 (d, J 16.8 Hz, 1H), 4.32 (d, J 16.8 Hz, 1H), 3.73 (s, 3H), 3.65 (s, 3H); ^13^C NMR (125.0 MHz, DMSO-d6) δ ppm: 182.9, 178.1, 157.0, 147.2, 146.2, 138.2, 135.4, 134.9, 134.0, 131.6, 131.0, 130.2, 128.4, 128.2, 127.1, 126.8, 126.7, 126.3, 124.6, 123.8, 56.7, 55.0, 53.8, 49.0. HR-ESIMS calc. for C_28_H_22_N_2_NaO_9_ [M + Na]^+^: 553.1217. Found: 553.1208. Δ 1.6 ppm.

Methylbenzyl((4-bromophenyl)(3-((methoxycarbonyl)oxy)-1,4-dioxo-1,4-dihydronaphthalen-2-yl)methyl)carbamate (**5d**). Light yellow solid, m.p. 155–156 °C, 63% yield. IR (KBr, cm^−1^): ν 2954, 1774, 1682, 1672, 1448, 1323, 1312, 1292, 1226, 1187, 1161, 1120, 947, 767, 700, 691, 683. ^1^H NMR (500.00 MHz, DMSO-d6) δ ppm: 7.92–7.81 (m, 4H), 7.47 (d, J 8.5 Hz, 2H), 7.20 (d, J 8.1 Hz, 2H), 6.94 (t, J 7.6 Hz, 2H), 6.87 (d, J 7.4 Hz, 2H), 6.82 (t, J 7.2 Hz, 1H), 6.78 (s, 1H), 4.88 (d, J 16.8 Hz, 1H), 4.35 (d, J 16.8 Hz, 1H), 3.71 (s, 3H), 3.69 (s, 3H). ^13^C NMR (125.0 MHz, DMSO-d6) δ ppm: 183.1, 178.2, 156.9, 150.9, 150.8, 138.5, 137.2, 135.3, 134.8, 134.8, 131.7, 131.6, 130.2, 129.5, 128.3, 127.0, 126.7, 126.5, 126.3, 120.8, 56.6, 55.1, 53.6, 48.8. HR-ESIMS calc. for C_28_H_22_BrNNaO_7_ [M + Na]^+^: 588.0471. Found: 588.0437. Δ 5.8 ppm.

Methylbenzyl((4-fluorophenyl)(3-((methoxycarbonyl)oxy)-1,4-dioxo-1,4-dihydronaphthalen-2-yl)methyl)carbamate (**5e**). Salmon solid, m.p. 124–126 °C dec., 76% yield. IR (KBr, cm^−1^): ν 2956, 1775, 1683, 1673, 1449, 1314, 1274, 1226, 1191, 1157, 943, 768, 700, 689. ^1^H NMR (500.00 MHz, DMSO-d6) δ ppm: 7.95–7.81 (m, 4H), 7.27 (dd, J 8.6 and 5.5 Hz, 2H), 7.08 (t, J 8.8 Hz, 2H), 6.97 (t, J 7.6 Hz, 2H), 6.87 (d, J 7.5 Hz, 3H), 6.78 (s, 1H), 4.77 (d, J 16.7 Hz, 1H), 4.41 (d, J 16.7 Hz, 1H), 3.72 (s, 3H), 3.68 (s, 3H). ^13^C NMR (125.0 MHz, DMSO-d6) δ ppm: 188.0, 183.0, 168.3, 165.1, 161.7, 155.7, 155.4, 143.4, 140.1, 140.0, 139.6, 138.2, 138.1, 136.4, 135.0, 134.6, 134.5, 133.1, 131.8, 131.5, 131.3, 131.1, 120.4, 120.1, 61.4, 60.0, 58.3, 53.5. HR-ESIMS calc. for C_28_H_22_FNNaO_7_ [M + Na]^+^: 526.1272. Found: 526.1273. Δ 0.2 ppm.

Methylbenzyl((3-((methoxycarbonyl)oxy)-1,4-dioxo-1,4-dihydronaphthalen-2-yl)(p-tolyl)methyl)carbamate (**5f**). Yellow solid, m.p. 163–164 °C, 67% yield. IR (KBr, cm^−1^): ν 2955, 1774, 1682, 1671, 1447, 1282, 1266, 1186, 1164, 1119, 1071, 942, 800, 769, 716. ^1^H NMR (500.00 MHz, DMSO-d6) δ ppm: 7.93–7.82 (m, 4H), 7.09 (d, J 3.8 Hz, 4H), 6.95 (t, J 7.5 Hz, 2H), 6.85 (d, J 7.6 Hz, 3H), 6.77 (s, 1H), 4.77 (d, J 16.2 Hz, 1H), 4.39 (d, J 16.7 Hz, 1H), 3.69 (s, 3H), 3.66 (s, 3H), 2.27 (s, 3H). ^13^C NMR (125.0 MHz, DMSO-d6) δ ppm: 183.4, 178.3, 156.9, 151.0, 150.5, 138.8, 137.1, 135.6, 135.3, 134.9, 131.6, 130.2, 129.5, 129.4, 128.3, 127.6, 127.0, 126.6, 126.5, 126.3, 56.6, 55.6, 53.4, 48.7, 20.9. HR-ESIMS calc. for C_29_H_25_ClNNaO_7_ [M + Na]^+^: 522.1523. Found: 522.1525. Δ 0.4 ppm.

Methylbenzyl((3-((methoxycarbonyl)oxy)-1,4-dioxo-1,4-dihydronaphthalen-2-yl)(4-methoxy phenyl)methyl)carbamate (**5g**). Brown solid, m.p. 150–152 °C dec., 55% yield. IR (KBr, cm^−1^): ν 2999, 2956, 2851, 1775, 1696, 1683, 1669, 1446, 1268, 1281, 1226, 1190, 1176, 1159, 1114, 940, 764, 717, 696, 686. ^1^H NMR (500.00 MHz, DMSO-d6) δ ppm: 7.96–7.85 (m, 4H), 7.14 (d, J 8.6 Hz, 2H), 6.99 (t, J 7.4 Hz, 2H), 6.89 (t, J 7.1 Hz, 1H), 6.85 (d, J 7.3 Hz, 2H), 6.81 (d, J 8.8 Hz, 2H), 6.71 (s, 1H), 4.63 (d, J 15.9 Hz, 1H), 4.44 (d, J 16.8 Hz, 1H), 3.73 (s, 3H), 3.72 (s, 3H), 3.64 (s, 3H). ^13^C NMR (125.0 MHz, DMSO-d6, TMS, δ ppm): 182.8, 177.7, 158.6, 150.6, 149.6, 138.3, 129.6, 127.7, 126.4, 126.1, 125.8, 125.7, 113.7, 56.1, 55.0, 52.9, 48.7, 47.9. HR-ESIMS calc. for C_29_H_25_ClNNaO_8_ [M + Na]^+^: 538.1472. Found: 538.1472. Δ 0 ppm.

Methylbenzyl((3-((methoxycarbonyl)oxy)-1,4-dioxo-1,4-dihydronaphthalen-2-yl)(m-tolyl) methyl)carbamate (**5h**). Deep yellow solid, m.p. 135–136 °C dec., 61% yield. IR (KBr, cm^−1^): ν 2956, 1779, 1674, 1448, 1271, 1225, 1192, 1120, 972, 949, 769, 739, 700, 689. ^1^H NMR (500.00 MHz, DMSO-d6) δ ppm: 7.93–7.89 (m, 2H), 7.87–7.82 (m, 2H), 7.18 (t, J 7.6 Hz, 1H), 7.06 (d, J 7.5 Hz, 1H), 7.02 (d, J 7.9 Hz, 1H), 6.99 (s, 1H), 6.95 (t, J 7.5 Hz, 2H), 6.85 (d, J 7.7 Hz, 3H), 6.80 (s, 1H), 4.79 (d, J 16.7 Hz, 1H), 4.40 (d, J 16.7 Hz, 1H), 3.68 (s, 6H), 2.21 (s, 3H). ^13^C NMR (125.0 MHz, DMSO-d6) δ ppm: 183.3, 178.2, 156.9, 150.8, 150.6, 138.9, 138.1, 135.3, 134.9, 131.6, 130.2, 128.7, 128.6, 128.4, 128.3, 127.5, 127.1, 127.0, 126.6, 126.5, 126.3, 124.3, 56.6, 53.5, 51.8, 48.7, 21.3. HR-ESIMS calc. for C_28_H_22_ClNNaO_7_ [M + Na]^+^: 522.1523. Found: 522.1515. Δ 1.5 ppm.

Methylbenzyl((3-chlorophenyl)(3-((methoxycarbonyl)oxy)-1,4-dioxo-1,4-dihydronaphthalen-2-yl)methyl)carbamate (**5i**). Light yellow solid, m.p. 150–152 °C, 81% yield. IR (KBr, cm^−1^): ν 2955, 1779, 1684, 1672, 1448, 1296, 1282, 1259, 1229, 1193, 1158, 1121, 946, 800, 701, 689. ^1^H NMR (500.00 MHz, DMSO-d6) δ ppm: 7.90–7.80 (m, 4H), 7.38–7.27 (m, 3H), 7.21 (m, 1H), 6.92 (t, J 7.5 Hz, 2H), 6.87 (d, J 7.2 Hz, 2H), 6.83 (s, 1H), 6.79 (t, J 7.2 Hz, 1H), 4.94 (d, J 16.8 Hz, 1H), 4.31 (d, J 16.7 Hz, 1H), 3.71 (s, 3H), 3.67 (s, 3H). ^13^C NMR (125.0 MHz, DMSO-d6) δ ppm: 183.0, 178.2, 150.9, 150.7, 140.4, 138.5, 135.3, 134.8, 134.5, 133.8, 131.6, 130.7, 130.2, 128.6, 128.3, 127.6, 127.1, 127.0, 126.7, 126.5, 126.3, 125.5, 56.7, 54.9, 53.7, 48.8. HR-ESIMS calc. for C_28_H_22_ClNNaO_7_ [M + Na]^+^: 542.0977. Found: 542.0954. Δ 4.2 ppm.

#### 2.1.3. General Procedures for Synthesis of Naphthoquinones **6**

A 50 mL round bottom flask containing a solution of lawsone (**1**, 5.7 mmol), arylaldehyde (**2**, 6.3 mmol), and benzylamine (**3**, 6.3 mmol) in dry THF (20 mL) under argon atmosphere was stirred for 1 h. Then, DBU (6.0 mmol) and methyl chloroformate (14.25 mmol) were added and the mixture was stirred overnight at room temperature. The solvent was removed under low pressure and the obtained residue was purified by column chromatography on silica gel using a hexane/ethyl acetate mixture as a gradient. The compounds **6a**–**i** were obtained as red solids in very good yields.

Methyl benzyl((4-chlorophenyl)(3-((methoxycarbonyl)oxy)-1,4-dioxo-1,4-dihydronaphthalen-2-yl)methyl)carbamate (**6a**). Brown solid, m.p. 108–110 °C, 80% yield. IR (KBr, cm^−1^): ν 3342, 3063, 3030, 2955, 1686, 1657, 1593, 1449, 1370, 1336, 1308, 1262, 1212, 730, 700, 532. ^1^H NMR (500.00 MHz, DMSO-d6) δ ppm: 10.01 (s, 1H), 7.84 (dd, J 7.7 and 0.9 Hz, 1H), 7.79 (d, J 7.6 Hz, 1H), 7.76 (td, J 7.5 and 1.3 Hz, 1H), 7.68 (td, J 7.5 and 1.3 Hz, 1H), 7.32 (d, J 8.5 Hz, 2H), 7.18 (d, J 8.1 Hz, 2H), 6.92–6.84 (m, 4H), 6.80 (m, 2H), 4.94 (d, J 16.9 Hz, 1H), 4.37 (d, J 16.8 Hz, 1H), 3.68 (s, 3H). ^13^C NMR (125.0 MHz, DMSO-d6) δ ppm: 192.2, 183.2, 183.1, 161.9, 140.9, 140.3, 135.4, 135.1, 134.8, 134.5, 133.5, 132.7, 131.5, 131.1, 130.1, 129.8, 129.1, 128.8, 128.7, 128.6, 127.9, 127.5, 127.1, 126.4, 126.3, 125.7, 122.9, 51.8, 44.5, 35.2. HR-ESIMS calc. for C_26_H_20_ClNNaO_5_^+^ [M + Na]^+^: 484.0922. Found: 484.0928. Δ 1.2 ppm.

Methyl benzyl((phenyl)(3-((methoxycarbonyl)oxy)-1,4-dioxo-1,4-dihydronaphthalen-2-yl)methyl)carbamate (**6b**). Red oil, 79% yield. IR (KBr, cm^−1^): ν 3065, 2359, 2336, 1661, 1593, 1573, 1369, 1281, 759, 731, 699, 576, 557, 531. ^1^H NMR (500.00 MHz, DMSO-d6) δ ppm: 10.03 (s, 1H), 7.97–7.93 (m, 1H), 7.85 (ddd, J 17.4, 7.6 and 0.7 Hz, 1H), 7.81–7.76 (m, 1H), 7.72 (ddd, J 5.9, 4.3 and 1.1 Hz, 1H), 7.29 (dt, J 17.1 and 8.3 Hz, 3H), 7.24–7.16 (m, 3H), 6.94–6.85 (m, 3H), 6.81 (t, J 7.0 Hz, 2H), 4.93 (d, J 16.8 Hz, 1H), 4.43 (d, J 16.8 Hz, 1H), 3.80 (s, 3H). ^13^C NMR (75.0 MHz, DMSO-d6) δ ppm: 193.4, 183.8, 182.0, 177.8, 156.7, 155.6, 141.4, 140.3, 134.9, 134.8, 133.2, 133.0, 130.6, 129.8, 129.5, 128.6, 128.4, 128.0, 127.4, 126.4, 125.9, 123.6, 54.3, 52.5, 48.1. HR-ESIMS calc. for C_26_H_21_NNaO_5_^+^ [M + Na]^+^: 450.13. Found: 450.41. Δ 1.9 ppm.

Methyl benzyl((4-nitrophenyl)(3-((methoxycarbonyl)oxy)-1,4-dioxo-1,4-dihydronaphthalen-2-yl)methyl)carbamate (**6c**). Yellow solid, m.p. 142–144 °C, 72% yield. IR (KBr, cm^−1^): ν 3321, 2957, 2857, 2455, 2362, 2326, 1687, 1656, 1595, 1517, 1449, 1375, 1308, 1291, 1262, 1211, 1118, 1044, 730, 701, 679, 653. ^1^H NMR (500.00 MHz, DMSO-d6) δ ppm: 8.06 (d, J 8.7 Hz, 2H), 7.79 (d, J 7.6 Hz, 1H), 7.66 (d, J 7.5 Hz, 1H), 7.61 (td, J 7.5 Hz, J 1.1 Hz, 1H), 7.47 (td, J 7.4 and 1.1 Hz, 1H), 7.33 (d, J 8.5 Hz, 2H), 6.99 (d, J 7.5 Hz, 2H), 6.93 (t, J 7.4 Hz, 3H), 6.85 (t, J 7.2 Hz, 1H), 4.80 (d, J 16.3 Hz, 1H), 4.67 (d, J 16.4 Hz, 1H), 3.57 (s, 3H). ^13^C NMR (75.0 MHz, DMSO-d6) δ ppm: 185.9, 178.3, 170.6, 156.6, 153.9, 144.8, 140.2, 135.3, 133.1, 131.0, 130.3, 129.7, 127.9, 126.9, 126.7, 126.4, 126.3, 125.7, 125.1, 124.3, 122.8, 122.4, 113.9, 55.9, 51.9, 48.1. HR-ESIMS calc. for C_26_H_20_N_2_NaO_7_^+^ [M + Na]^+^: 495.1163. Found: 495.1138. Δ 5.0 ppm.

Methyl benzyl((4-bromo-phenyl)(3-((methoxycarbonyl)oxy)-1,4-dioxo-1,4-dihydronaphthalen-2-yl)methyl)carbamate (**6d**). Orange solid, m.p. 150–152 °C dec., 75% yield. IR (KBr, cm^−1^): ν 3355, 1687, 1656, 1449, 1382, 1336, 1309, 1291, 1264, 730, 701, 681, 611. ^1^H NMR (500.00 MHz, DMSO-d6) δ ppm: 7.83 (d, J 7.7 Hz, 1H), 7.76 (d, J 7.5 Hz, 1H), 7.71 (t, J 7.5 Hz, 1H), 7.61 (t, J 7.4 Hz, 1H), 7.42 (d, J 8.3 Hz, 2H), 7.09 (d, J 8.0 Hz, 2H), 6.90 (d, J 3.9 Hz, 4H), 6.81 (sl, 2H), 4.88 (d, J 16.4 Hz, 1H), 4.48 (d, J 16.2 Hz, 1H), 3.64 (s, 3H). ^13^C NMR (75.0 MHz, DMSO-d6) δ ppm: 184.1, 182.6, 174.9, 157.3, 141.8, 141.0, 134.1, 133.9, 132.2, 131.6, 130.9, 130.7, 129.7, 128.5, 127.7, 126.4, 126.2, 125.8, 125.5, 122.5, 118.1, 55.9, 52.7, 51.6, 49.1, 48.9, 45.5, 38.7, 37.1, 33.4, 29.6, 28.8, 28.7, 23.5. HR-ESIMS calc. for C_26_H_20_BrNNaO_5_^+^ [M + Na]^+^: 528.0417. Found: 528.0406. Δ 2.1 ppm.

Methyl benzyl((4-fluor-phenyl)(3-((methoxycarbonyl)oxy)-1,4-dioxo-1,4-dihydronaphthalen-2-yl)methyl)carbamate (**6e**). Red oil, 72% yield. IR (KBr, cm^−1^): ν 3335, 3065, 3031, 2924, 2853, 1700, 1671, 1649, 1524, 1508, 1363, 1338, 1257, 1158, 1043, 726, 696, 523. ^1^H NMR (500.00 MHz, DMSO-d6) δ ppm: 7.80 (dd, J 7.7 and 0.9 Hz, 1H), 7.66 (dd, J 7.6 and 0.9 Hz, 1H), 7.60 (td, J 7.5 and 1.3 Hz, 1H), 7.45 (td, J 7.5 and 1.3 Hz, 1H), 7.09 (dd, J 8.2 and 5.7 Hz, 2H), 7.00 (d, J 7.2 Hz, 2H), 6.98–6.91 (m, 4H), 6.89–6.84 (m, 2H), 4.81 (d, J 16.3 Hz, 1H), 4.70 (d, J 16.3 Hz, 1H), 3.54 (s, 3H). ^13^C NMR (75.0 MHz, DMSO-d6) δ ppm: 187.9, 179.8, 171.0, 162.2, 159.0, 157.4, 141.2, 134.2, 130.6, 128.6, 128.0, 127.9, 127.6, 127.5, 127.1, 126.4, 125.9, 125.8, 125.0, 115.7, 114.5, 114.2, 69.9, 55.9, 52.6, 51.8, 49.0, 44.5. HR-ESIMS calc. for C_26_H_20_FNNaO_5_^+^ [M + Na]^+^: 468.1218. Found: 468.1212. Δ 1.3 ppm.

Methyl benzyl((3-methyl-phenyl)(3-((methoxycarbonyl)oxy)-1,4-dioxo-1,4-dihydronaphthalen-2-yl)methyl)carbamate (**6h**). Red oil, 92% yield. IR (KBr, cm^−1^): ν 3071, 1660, 1633, 1594, 1575, 1349, 1282, 968, 915, 828, 787, 729, 708, 666, 575. ^1^H NMR (500.00 MHz, DMSO-d6) δ ppm: 7.80 (d, J 7.6 Hz, 1H), 7.68 (d, J 7.6 Hz, 1H), 7.62 (t, J 7.5 Hz, 1H), 7.47 (t, J 7.5 Hz, 1H), 7.30 (d, J 7.4 Hz, 1H), 7.25 (d, J 7.4 Hz, 1H), 7.05 (t, J 7.5 Hz, 1H), 7.00 (d, J 7.5 Hz, 2H), 6.94 (t, J 7.5 Hz, 2H), 6.88–6.85 (m, 3H), 4.84 (d, J 16.3 Hz, 1H), 4.71 (d, J 16.3 Hz, 1H), 3.53 (s, 3H), 2.20 (s, 3H). ^13^C NMR (75.0 MHz, DMSO-d6) δ ppm: 187.0, 178.9, 170.3, 156.7, 143.9, 140.6, 135.7, 135.4, 133.4, 131.4, 130.8, 129.7, 127.9, 126.9, 126.8, 126.7, 126.3, 125.7, 125.1, 124.9, 124.3, 122.8, 115.4, 55.6, 51.8, 48.5, 20.9. HR-ESIMS calc. for C_27_H_23_NNaO_5_^+^ [M + Na]^+^: 464.1468. Found: 464.1458. Δ 2.1 ppm.

Methyl benzyl((3-chloro-phenyl)(3-((methoxycarbonyl)oxy)-1,4-dioxo-1,4-dihydronaphthalen-2-yl)methyl)carbamate (**6i**). Red oil, 75% yield. IR (KBr, cm^−1^): ν 3332, 2945, 1593, 1571, 1360, 1275, 1215, 1159, 970, 783, 727, 690, 525. ^1^H NMR (500.00 MHz, DMSO-d6) δ ppm: 7.79 (d, J 7.6 Hz, 1H), 7.66 (d, J 7.3 Hz, 1H), 7.61 (t, J 7.4 Hz, 1H), 7.47 (t, J 7.3 Hz, 1H), 7.21 (t, J 7.7 Hz, 1H), 7.10 (d, J 7.6 Hz, 1H), 7.05–7.02 (m, 2H), 6.98 (d, J 7.1 Hz, 2H), 6.93 (t, J 7.0 Hz, 2H), 6.86–6.85 (m, 2H), 4.73 (s, 2H), 3.56 (s, 3H). ^13^C NMR (75.0 MHz, DMSO-d6) δ ppm: 179.1, 156.8, 147.1, 140.3, 135.2, 133.6, 133.5, 132.5, 132.3, 131.7, 130.9, 130.1, 129.1, 128.1, 127.1, 126.9, 126.6, 125.7, 125.3, 125.3, 125.0, 124.5, 124.2, 55.4, 52.2, 48.3. HR-ESIMS calc. for C_26_H_20_ClNNaO_5_^+^ [M + Na]^+^: 484.0922. Found: 484.0924. Δ 0.4 ppm.

### 2.2. Biological Assays

Cells and reagents. Human SCC-4, SCC-9 and SCC-25 cells, derived from a human oral tongue SCC were obtained from the ATCC (CRL-1624; CRL-1629 and CRL-1628, respectively) and maintained at 1:1 DMEM/F12 (Dulbecco’s modified Eagle medium and Ham’s F12 medium; Gibco, Thermo Fisher, Waltham, MA, USA) supplemented with 10% (*v/v*) FBS (fetal bovine serum; Invitrogen, Thermo Fisher, Waltham, MA, USA) and 400 ng/mL hydrocortisone (Sigma-Aldrich Co., St. Louis, MO, USA). Primary normal human gingival fibroblasts were obtained from the ATCC (PCS-201-018), maintained in DMEM supplemented with 10% (*v/v*) FBS, and used in a maximum of 6 passages. Cells were grown in a humidified environment containing 5% CO_2_ at 37 °C. For all biological experiments: compounds and controls lapachol, shikonin, and doxorubicin were solubilized in 100% DMSO (Sigma-Aldrich) to a final concentration of 10 mM. Carboplatin in water (Fauldcarbo^®^; Libbs Farmacêutica, São Paulo, SP, Brazil) was used as a standard anticancer compound.

Cell viability assay (cytotoxicity) and stability assay. To assess cell viability, cells were grown in triplicate in 96-well plates (5 × 10^3^ cells/well) until confluence when treated with the different compounds as in Machado et al. [11]. Briefly, DMSO in the same concentrations was used as a 100% cell viability control. After 48 h, cells were incubated with 5 mg/mL MTT reagent (3-(4,5-dimethyl-2-thiazolyl)-2,5-diphenyl-2*H*-tetrazolium bromide) (Sigma-Aldrich Co., St. Louis, MO, USA) for 3.5 h. Formazan crystals were dissolved in MTT solvent (DMSO/methanol 1:1 *v/v*) and the absorbance at 560 nm was evaluated using an EPOCH microplate spectrophotometer (BioTek Instruments, Winooski, VT, USA) with background absorbance at 670 nm subtracted. Each of the eighteen compounds was tested at six or seven different concentrations, ranging from 0.3 µM to 200 µM in cancer cell lines (SCC-4, SCC-9 and SCC-25) and 0.4 µM to 300 µM in normal primary human gingival fibroblast. Controls (carboplatin, lapachol, shikonin, and doxorubicin) were tested in six or seven different concentrations ranging from 5 µM to 1000 µM. For assays using cell death inhibitors, cells were incubated with ZVAD (Sigma-Aldrich Co., St. Louis, MO, USA); Necrostatin-1 (Sigma-Aldrich Co., St. Louis, MO, USA) at 50 µM for 2 h, and then treated with 1 × IC_50_ of compound **6a** or control (DMSO) for an additional 24 h, for 3-MA at 5000 µM for 2 h, and then treated with 2 × IC_50_ of compound **6a** or control (DMSO and shikonin).

For the stability test, compound **6a** and controls (carboplatin and doxorubicin) were prepared at concentration of 2 × IC_50_ and stored in the incubator at 37 °C for 0, 1, 3, 6, 12, 24 or 48 h before SCC9 cell treatment (5 × 10^3^ cells/well). After the desired treatment time was reached, the medium containing the compound was replaced with untreated culture medium and the MTT assay was performed at the end of both experiments.

Hemolysis assay. Human blood was used as approved by the Research Ethics Committee of the Fluminense Federal University—Nova Friburgo-RJ (CAAE: 43134721.4.0000.5626). The erythrocytes were collected by centrifugation at 1500 rpm for 15 min, washed with PBS (phosphate buffer saline) with 10 mM glucose and counted in an automatic cells counter (Thermo Fisher, Waltham, MA, USA). The erythrocytes were then plated in 96-well plates, at a concentration of 4 × 10^8^/well in triplicates, and 10 µL of the compounds added at a final concentration of 400 µM in PBS with glucose (final volume of 100 µL). 10 µL of PBS were used as negative control and 10 µL of PBS with 0.1% Triton ×100 as a positive control. Data reading was performed with EPOCH (BioTek Instruments, Winooski, VT, USA) at 540 nm absorbance and statistical data were generated with the GRAPHPAD Prism 5.0 program (Intuitive Software for Science, San Diego, CA, USA).

Clonogenic assay. The SCC9 strain and primary human fibroblasts were grown in duplicates in 24-well plates (5 × 10^4^ cells/well) and incubated for 24 h. Then, they were treated with the most selective compounds with 2 × IC_50_. DMSO was used at the same concentrations as a control for 100% of viability. After 5 days of incubation, the culture medium was removed and the cells were fixed with absolute ethanol (500 µL) for 10 min, then stained with crystal 0.05% violet (Sigma Aldrich) in 20% ethanol (500 µL) for 10 min and washed with distilled water. The plates were scanned to quantify the inhibition of the formation of colonies. Then, crystal violet was solubilized in 33% acetic acid and added to the 96-well plate. Finally, the absorbance was read in a spectrophotometer (EPOCH, BioTek Instruments, Winooski, VT, USA) (595 nm).

In vivo acute toxicity study. The acute toxicity study for compound **6a** was performed as in the work of Macedo et al. [12]. Briefly, the assay was carried out with C57BL/6 female mice at twelve weeks of age via intraperitoneal injection and was approved by the University Animal Ethics Board under registration number 982. All experiments were performed in accordance with Brazilian guidelines and regulations. Dosing and analysis were performed according to OECD guidelines 423 and revised by Parasuraman [13]. Each animal group had *n* = 3 and received only one intraperitoneal injection (Day 0) of compound **6a** dissolved in 3 mL of PBS and 3% of DMSO. Control group animals received only 3% DMSO in PBS. The first dose of the compound was 100 mg/kg. Subsequent dose levels (150 mg/kg and 200 mg/kg) were determined based on the result obtained in the previous dosage. The animals were examined every day, twice a day, for the mortality and morbidity for 14 days, when all animals were anesthetized (ketamine 100 mg/kg and xylazine 10 mg/kg) and sacrificed by cervical dislocation followed by gross necropsy and histology of the main organs. The animals’ body weight and average food consumption were measured every 7 days as an indication of morbidity, and the following signs were assessed: tremors; convulsion; salivation; diarrhea; lethargy; pain signs; increased rear arching and defect in mobility. The necropsy included an examination of the external characteristics of the carcass; external body orifices; the abdominal, thoracic, and cranial cavities; organs/tissues liver, thymus, right kidney, right testicle, heart, and lung.

Video microscopy. SCC-9 cells previously plated the day before the experiment, were treated with 2 × IC_50_ of compound **6a** and transferred to a culture chamber adapted to a Nikon Eclipse TE300 microscope (Nikon, Melville, NY, USA) under controlled conditions of CO_2_ and temperature (5% and 37 °C, respectively). For 72 h, phase contrast images of the same field were captured every minute using a Hamamatsu C2400 CCD camera (Hamamatsu, Japan). The same experimental conditions were used for control (DMSO). The images of each experimental condition were integrated into videos using ImageJ software (National Institute of Health, Bethesda, MD, USA).

Autophagy assay. Cells of the SCC9 lineage were plated in 96-well plates (5 × 10^3^ cells/well). After 24 h, they were treated with 2 × IC_50_ of compound **6a** or with the controls shikonin, DMSO or serum-free medium. 12 h before labeling, half of the wells were treated with the autophagy inhibitor 3-Methyladenine (5 mM) (Sigma). For labeling at the indicated times, the DMEM medium was removed from the wells, replaced with 100 µL of the autophagosome marker (Autophagy Assay KIT, MAC138-Sigma-Aldrich), and then incubated for 30 min in the incubator (37 °C and 5% CO_2_). After this time, the cells were washed and photographed in an inverted fluorescence Zeiss microscope for 16 h for Shikonin and 36 h for compound **6a**.

ROS production. The hydrogen peroxide (H_2_O_2_) luminescence detection assay was performed using the ROS-Glo™ H_2_O_2_ assay kit (Promega, Madison, WI, USA). To perform the experiment, 1 × 10^5^ SCC9 cells were plated in DMEM F12 10% FBS medium per well in a 96-well plate and, after 24 h of incubation, the medium was removed and wells treated with 2 × IC_50_ of compound **6a** in SCC9, or with the controls DMSO and doxorubicin. Cell-free wells were used as a control. Menadione (M9529-Sigma-Aldrich) was used as a positive control. After the indicated time, the H_2_O_2_ substrate was used for all treatments, and the plate was incubated for the remaining 2 h. After 3, 6, and 24 h, the detection solution was added and the plates with the samples remained at room temperature for 20 min, followed by reading using the luminometer (Turner Designs, TD 2020, San Jose, CA, USA).

Cell cycle and SubG1 analysis. To evaluate the action of compound **6a** on the cell cycle, cells of the SCC9 lineage were plated in a 6-well plate (5 × 10^5^ cells/well) as described in [14]. After 48 h of treatment, cells were trypsinized and stained with propidium iodide (75 µM Sigma) in the presence of NP-40 (Sigma). DNA content was analyzed by collecting 1 × 10^4^ events using a FACScalibur flow cytometer. The data were analyzed with CellQuest (BD Biosciences, Franklin Lakes, NJ, USA) and FlowJo (Tree Star Inc., Ashland, OR, USA) software.

Effector caspases activation and Phosphatidyl serine exposure analysis (apoptosis). Cells of the SCC9 lineage were plated in 6-well plates (5 × 10^5^ cells/well), trypsinized 48 h after treatment, labeled using the Annexin V-FITC Apoptosis detection Kit according to the manufacturer’s protocol (BMS500FI, Invitrogen) and analyzed by cytometry flow as described in [15]. Furthermore, 5 × 10^4^ SCC9 cells were plated in a 24-well plate containing 1 mL of DMEM/F12 with 10% FBS per well. CellEvent™ Caspase-3/7 Reagent (#R37111, Invitrogen) was diluted in culture medium according to the manufacturer’s instructions. Hence, 24 h after plating, cells were treated with Caspase-3/7 Reagent and 2 × IC_50_ of substance **6a** or DMSO as a control. Cells were analyzed by flow cytometry at the time of 48 h of treatment.

Statistical analysis, IC_50_ calculation. IC_50_ values for MTT assays were obtained by non-linear regression using the GraphPad Prism 5.0 software (GraphPad, San Diego, CA, USA) from at least three independent experiments. Data are presented as means ± SD. A dose-response (inhibitor) vs response curve using the least squares method was used to determine the IC_50_, SD and R2 of the data. The selectivity index was calculated as S.I. = IC_50_ of the compound in normal oral fibroblast cells/IC_50_ of the same compound for each oral cancer cell line (SCC-4, SCC-9 and SCC-25) and the mean was calculated when indicated.

### 2.3. In Silico Studies

Molecular docking analysis. A reverse docking approach was employed to evaluate the potential targets of the most promising compound 6a and further provide a putative mechanism of action. Five proteins known as targets of lapachol, and other naphthoquinones were chosen: DNA-binding domain of topoisomerase I (PDB code 1K4T), IIα (PDB code 5GWK) and IIβ (PDB code 3QX3), ATPase domain of topoisomerase IIα (PDB code 1ZXM), human pyruvate kinase M2 (hPKM2, PDB code 3SRD). The three-dimensional structures of **6a**, shikonin, and lapachol were constructed and optimized using the Spartan’10 software and molecular docking studies were carried out using Autodock Tools 1.5.7 and Autodock Vina 1.1.2 [16] as described elsewhere [17,18]. The conformation of the ligands with the lowest binding energy with each enzyme was analyzed using Discovery Studio Visualizer 2019 (Dassault Systèmes BIOVIA, San Diego, CA, USA, 2019) and Pymol v. 1.2r2 (ThePyMOL Molecular Graphics System, Version 1.2r2 Schrödinger, LLC, New York, NY, USA). Since **6a** was assayed as a racemic mixture, we docked both *R* and *S* enantiomers. According to their binding modes, our results suggested that the enantiomer *R* contributes mostly to the cytotoxic activity. Thus, we focused our analysis on this enantiomer.

Prediction of toxicity and pharmacokinetic properties. The Smile structure of the compounds evaluated was obtained using the ChemDraw software (https://www.perkinelmer.com/category/chemdraw, accessed on 3 August 2022). The values of calculated octanol-water partition coefficient (cLogP), molecular weight (MW), number of hydrogen bond acceptors (nON), number of hydrogen bond donors (nOH/NH), and topological polar surface area (TPSA) were calculated using the SwissADME web server (http://www.swissadme.ch/, accessed on 3 August 2022). Other predictions, such as absorption, distribution, and metabolism, were performed with the admetSAR 2.0 server (http://lmmd.ecust.edu.cn/admetsar2, accessed on 3 August 2022). The most selective compound was analyzed, and carboplatin and doxorubicin were used as controls.

## 3. Results and Discussion

### 3.1. Chemistry

**Scheme 1:**
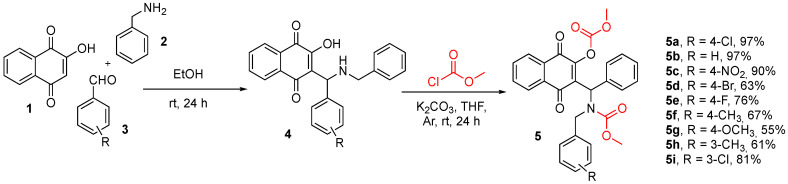
Synthesis of naphthoquinone derivatives **5a**–**i**.

To obtain the naphthoquinone derivatives **5**, it was initially necessary to synthesize the Mannich’s bases **4**, through a reaction involving three components (3-MC), namely lawsone (**1**), aromatic aldehyde (**3**), and benzylamine (**2**) (Scheme 1). All bases were obtained as red solids in good yields as we described in previous work [19,20,21]. Then, the bases were reacted with methylchloroformate, potassium carbonate using dry THF as solvent at room temperature during 24 h, resulting in compounds **5** in good yields in the range of 55–97% (Scheme 1).

The structures of the compounds were elucidated by spectroscopic techniques (see Section 2 and Supplementary Materials). In the ^1^H NMR spectrum analysis of compound **5b**, two singlets could be observed at 3.68 and 3.67 ppm for methyl of carbamate and carbonate groups and two doublets at 4.83 ppm (d, *J* 16.8 Hz, 1H) and 4.40 ppm (d, *J* 16.8 Hz, 1H) corresponding to hydrogens methylene of the benzyl group. The two signals at 183.3 and 178.2 ppm in ^13^C RMN corresponding to carbonyl groups confirm the presence of the naphthoquinone moiety.

**Scheme 2:**
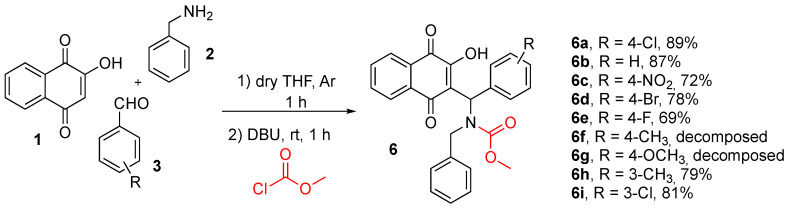
Synthesis of naphthoquinone derivatives **6a**–**i**.

On the other hand, any attempt to obtain a monoalkylated derivative where we varied the stoichiometry of the K_2_CO_3_ base and/or the methylchloroformate, or even reducing the reaction time, led to the recovery of starting material **4** or the formation of product **6**. However, when performing the three-component Mannich reaction followed by addition of DBU base and methylchloroformate in a one-pot reaction, after one hour at room temperature, we obtained the monoalkylated compounds of type **6a**–**i** as a red solid in good yields that in the range of 69–89% (Scheme 2).

The structures of the compounds were elucidated by spectroscopic techniques (see Section 2 and Supplementary Materials). In the ^1^H NMR spectrum analysis of compound **6a**, two singlets could be observed at 10.01 and 3.68 ppm for free hydroxyl group and methyl of carbamate group, respectively, confirming the monoalkylation, and two doublets at 4.94 ppm (d, *J* 16.9 Hz, 1H) and 4.37 ppm (d, *J* 16.8 Hz, 1H) corresponding to hydrogens methylene of benzyl group. The two signals at 192.2 and 183.2 ppm in ^13^C RMN corresponding to carbonyl groups confirm the presence of the naphthoquinone moiety.

**Scheme 3:**
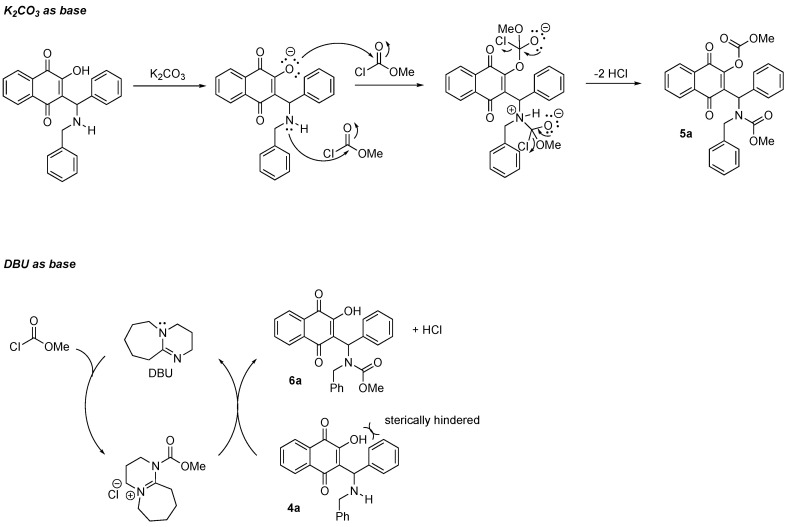
Proposal of intermediaries for the formation of **5a** and **6a**.

We believe that the nature of the base is responsible for directing the production of mono (**5a**–**i**) or dialkylated (**6a**–**i**) products, i.e., small bases, such as K_2_CO_3_, lead to the formation of products **5a**–**i**, and bulky bases, such as DBU, lead to the formation of products **6a**–**i**. For this, we carry out the alkylation reaction of product **4a**, e.g., by treatment with DBU and methylchloroformate in dry THF as solvent, and we observe the formation of only compound **6a**. Thus, based on such experimental evidence, we propose the sequence of intermediates in Scheme 3.

All compounds were characterized by physical methods of analysis, such as ^1^H and ^13^C NMR, high resolution mass spectrometry and spectroscopy in the infrared region (See Supplementary Materials).

### 3.2. Biological Activity

#### 3.2.1. Cytotoxicity and Selectivity, Hemolytic Potential, and Stability of the New Compounds

Initially, the sixteen Mannich adducts (**5a**–**i**, **6a**–**e**, **6h**–**i**) were submitted to the MTT assay. First, the assay was performed on the SCC9 oral cancer cell lineage and as controls we used known chemotherapeutic agents, namely carboplatin, gold standard for the treatment of oral cancer [22,23], doxorubicin, a naphthoquinone used in the treatment of some cancers [24,25], shikonin and lapachol, naphthoquinones with antitumor potential well described in the literature [26,27]. Of the sixteen compounds tested, all of them showed dose-dependent cytotoxicity and were subsequently tested on untransformed primary human gingival fibroblasts (Table 1). Anticancer activities of these compounds are reported here for the first time.

The degree of selectivity of these compounds can be expressed by their selective index (SI). A high SI value ≥ 2 of a compound represents a selective toxicity towards cancer cells, while a SI value < 2 is considered generally toxic, such that it can also cause cytotoxicity in normal cells. We observed that compounds **6a** (IS: 3.31) and **6h** (IS: 4.83) were the most selective (Table 1, highlighted), being superior to other naphthoquinones already tested against OSCC in the literature [28,29]. These naphthoquinones derivatives mentioned were even more selective than the controls.

The initial screening was performed with the SCC9 cell line because it is more sensitive to antitumor agents. As selectivity is one of the main factors that assess the effectiveness of chemotherapeutic agents, it becomes important to consider other oral cancer cell lines, so compounds with an S.I. above 3 were selected for testing in two more tumor cell lines (SCC4 and SCC25). We found that among the three most selective compounds, only **6a** maintained the selectivity index above 2 with an IS of 2.36, surpassing carboplatin (IS 0.847) and lapachol (IS 1.66). Furthermore, doxorubicin (IS 3.39) was the unique control that showed selectivity, but it is not commonly used to treat oral cancer. Only compound **6a** was used for the following assays since it presented selectivity on average against all OSCC cell lines tested (Table 2).

**Figure 1:**
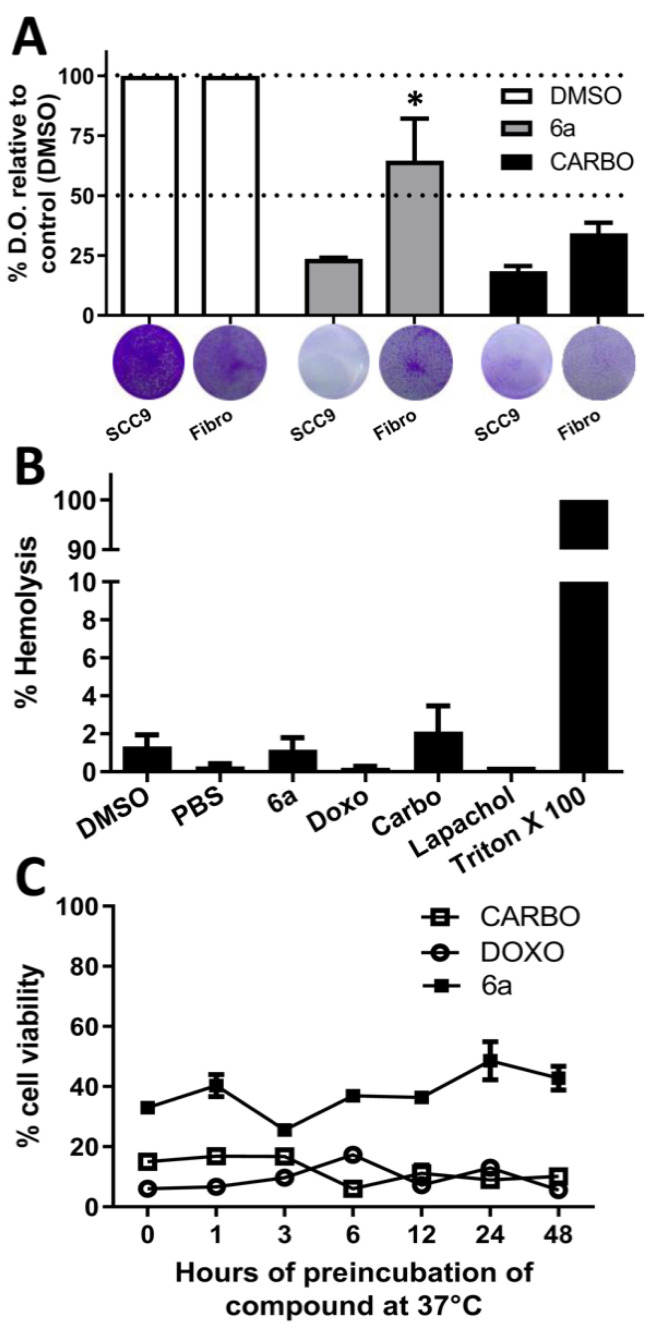
Clonogenic, hemolytic and stability assays. (**A**) Clonogenic assay: each column indicates the cell type (SCC-9 or normal fibroblast) treated with the indicated compound. Cells were treated with twice the calculated IC_50_ (2 × IC_50_) in SCC-9 cells (**6a**: 112.48 µM). Top: graphical analyses of colony formation by absorbance reading as indicated in material and methods. Bottom: representative wells of colony formation assay stained with crystal violet. One-way ANOVA with Boferroni’s multiple comparison test was performed where all columns were significantly different from the control (DMSO) and * represents *p* < 0.0001. (**B**) Hemolytic activity of compounds at 400 μM (approximately 10 × IC_50_). One-way ANOVA with Dunnett’s Posttest was performed where all columns were significantly different from the control (Triton X) with *p* < 0.0001. (**C**) The stability of compound **6a** was evaluated. The compound and the controls were diluted to 2 × IC_50_ and incubated in the incubator at 37 °C for 1/3/6/12/24/48 h prior to SCC9 cells treatment for more 48 h. As controls, Carboplatin, Doxorubicin and DMSO were used.

Supporting the MTT assay, which indirectly correlates with cell viability, a clonogenic assay was performed, which directly quantifies the accumulation of cells by a given treatment. The clonogenic assay assesses the ability of a cell subjected to a particular treatment to self-duplicate and form a colony [30]. Cells of the SCC9 lineage and human primary fibroblasts were treated with the equivalent of two times the value of IC_50_ (2 × IC_50_ 112.48 µM) of compound **6a** and the carboplatin control. Both compound **6a** and carboplatin showed high cytotoxicity in the tumor cell line, while in fibroblasts, treatment with compound **6a** induced a low cytotoxicity in these normal cells (Figure 1A), in which more than 70% of the cells were viable and able to form colonies. With carboplatin, less than 50% of these normal cells maintained their ability to form colonies. These results reinforce that compound **6a** presents good selectivity, being more cytotoxic to tumor cells than in normal cells in a more efficient manner than carboplatin, corroborating the MTT results.

Furthermore, to discard any surfactant activity of the compounds, which could lead to unspecific cytotoxicity through cellular membrane damage, a hemolytic assay was performed and showed that compound **6a** and controls do not have hemolytic potential, showing less than 3% hemolysis compared to positive control, triton X-100, which represents 100% of cell lysis in red blood cells (Figure 1B). Studies in the literature show that other synthetic naphthoquinones also lack hemolytic potential [18,28,29,31]. Together, these data indicate that compound **6a** is selective against oral cancer tumor cells and non-hemolytic, making in vivo testing possible.

To be considered a good drug, the compound must have relative stability at 37 °C comparable to drugs already in use. To assess the stability of the new compound, **6a** and the controls were pre-incubated for different time intervals at 37 °C (0, 1, 3, 6, 12, 24, and 48 h) at a concentration of 2 × IC_50_ to later be tested for 48 h for their cytotoxicity against SCC9 cells. The results indicated that all three compounds, **6a**, carboplatin, and doxorubicin, are stable even after 48 h of pre-incubation at 37 °C (Figure 1C) with little variation in the cytotoxicity induced by them.

#### 3.2.2. Acute Toxicity In Vivo

Pre-clinical tests in animals are very important for drug development and for understanding the therapeutic potential of new molecules [13]. Thus, compound **6a** was submitted to the acute toxicity test in C56BL/6 mice to study its toxic potential. The first group of animals received the dose of 100 mg/kg with no changes in morbidity and mortality (Table 3). Afterwards, 200 mg/kg was tested in another group where changes such as decreased activity and back-archingin animals, which can be a sign of pain, were observed. In this group, all the animals died on the 6th day after treatment. To establish a dose that is lethal in 50% of the animals (LD_50_) [32], we tested 150 mg/kg in four animals. Morbidity changes, such as decreased activity, were observed in two animals and blindness of one eye was observed in one animal. The other two animals remained normal during the role experiment. Seven days after treatment the two animals that presented morbidities died and the others remained alive and normal until the 14th day, suggesting that the LD_50_ is close to 150 mg/kg (Table 3), a value comparable to that found for carboplatin in animal tests [33]. There was no significant difference in body weight between living animals at the end point (Supplementary Material). The results of the histopathological analysis shown in Table 3 indicate that the 100 mg/kg, 150 mg/kg, and 200 mg/kg groups demonstrated moderate portal hyperemia and intracytoplasmic vacuolar degeneration in the liver, while in the kidneys few signs of renal damage were found by observation of hyperemia, at this point. A similar result has already been reported in the literature with derivatives of 2-hydroxy-3-anilino-1,4-naphthoquinones [31]. Furthermore, in the 150 mg/kg and 200 mg/kg groups, discrete signs of a possible inflammation in the lungs were observed due to the presence of a focus of peribronchial lymphocyte infiltrate that were absent in the lower dose group.

#### 3.2.3. Molecular Docking of Compound **6a**

The enzymes Topoisomerases I and Topoisomerase II act on DNA replication and chromosome segregation. Topoisomerase II is an important therapeutic target of anticancer and antibacterial agents [34,35]. To obtain more information about the mechanism of action of compound **6a** responsible for the cytotoxic effects, we investigated whether this compound could bind to these enzymes, which are targeted by lapachol and other naphthoquinone derivatives [36,37,38,39,40]. The controls lapachol, topotecan, and etoposide, known inhibitors of topoisomerases I and II, respectively, were compared to **6a** regarding the binding mode to these enzymes. In this session the results will shortly be presented. The detailed analysis of compound **6a** and its possible targets can be found in the Supplementary Material.

**Figure 2:**
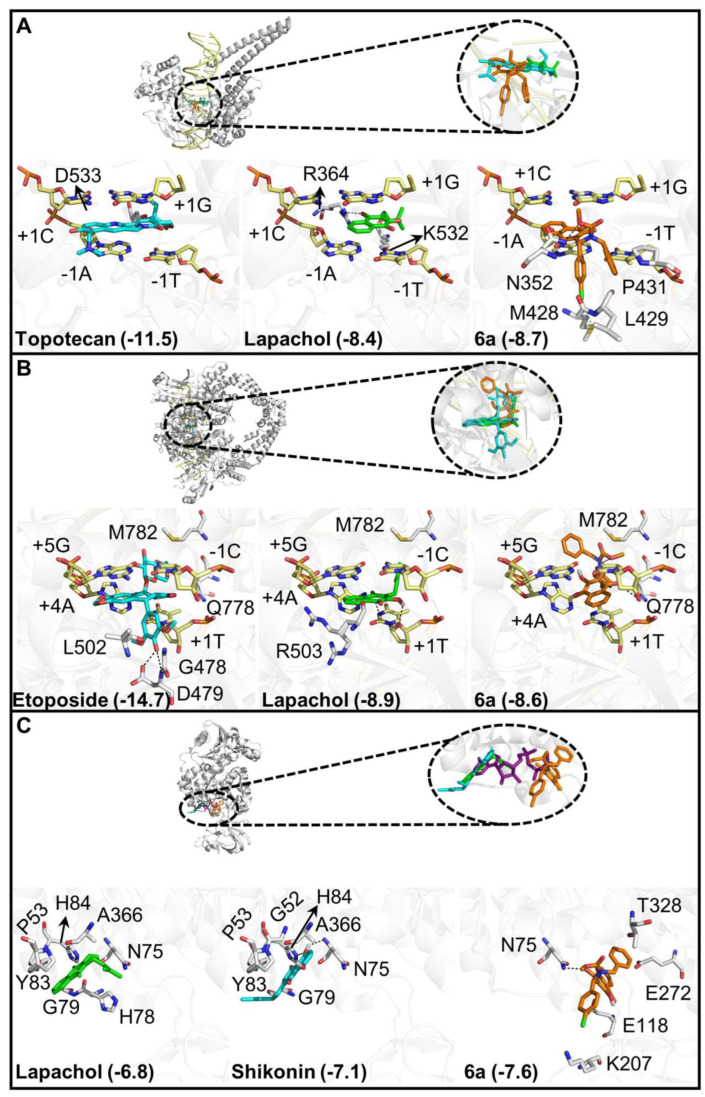
Prediction of the binding mode of **6a** with potential targets and comparison with lapachol, shikonin and co-crystallized inhibitors. Interaction profile and superimposition of (**A**) topotecan (cyan), lapachol (green), and **6a** (orange) complexed with the DNA-binding domain of topoisomerase I; (**B**) etoposide (cyan), lapachol (green), and **6a** (orange) in complex with the DNA-binding domain of topoisomerase IIβ; and (**C**) lapachol (green), shikonin (cyan), and **6a** (orange) complexed with the hPKM2. ATP (purple) bound to the Rabbit PKM2 (93.21% sequence identity) was also superimposed with lapachol, shikonin, and **6a** for comparison purposes. The carbon atoms of the amino acid residues and DNA bases are colored in white and yellow, respectively. Hydrogen bonds are shown as black dashed lines, while halogen bonds are shown as green dashed lines. The predicted binding energy (in Kcal/mol) of each ligand is shown in parentheses (binding energy values of the co-crystallized inhibitors were obtained by redocking). For more information and results, see Supplementary Materials.

Molecular docking studies with topoisomerase I (Figure 2A) indicated that lapachol intercalates between the base pairs +1C/+1G and −1T/−1A, as does the inhibitor topotecan, while its aliphatic chain binds to the minor groove of DNA as previously shown by Cavalcanti et al. [28]. Unlike topotecan and lapachol, 6a exploits several amino acid residues in the enzyme, which can help stabilize its binding pose. The binding energy of the 6a complex (−8.7 Kcal/mol) with topoisomerase I was similar to that of lapachol (−8.4 Kcal/mol), and higher than that of topotecan (−11.5 Kcal/mol). In the case of the topoisomerase IIβ, this compound explored interactions similar to those observed for lapachol and etoposide, but the binding mode was different. The binding energy of 6a (−8.6 Kcal/mol) was higher than etoposide (−14.7 Kcal/mol) and similar to lapachol (−8.9 Kcal/mol) (Figure 2B), indicating that compound 6a has lower binding affinity with Topoisomerase I and II enzymes than the chemotherapeutic controls already known as inhibitors and comparable to lapachol, reinforcing the potential of this compound.

However, the main anticancer activity of this new naphthoquinone derivate may arise from the inhibition of targets other than topoisomerases. The PKM2 protein plays an important role in cancer cell energy metabolism, in addition to being able to act as a transcription cofactor for various types of oncogenes. The overexpression of PKM2 is also related to multidrug resistance, metastasis, and angiogenesis [41]. It has been demonstrated that lapachol is able to impair the metabolism of cancer cells by targeting the PKM2 enzyme [42], as well as shikonin, another naphthoquinone [43,44]. In our docking studies, both lapachol and shikonin (lapachol, −6.8 Kcal/mol; shikonin, −7.1 Kcal/mol) (Figure 2C) occupied the entrance of the ATP binding cavity, an allosteric inhibition site of the enzyme. Although 6a was not superimposed with lapachol or shikonin, it still bound to the ATP binding cavity in a deeper region with apparent higher affinity (−7.6 Kcal/mol) than these controls and explored a more complex region, where the phosphate groups of ATP bind to a homologous enzyme [45]. These results correlate with the selectivity determined for these compounds in OSCC cells and indicate a possible mechanism of action. New drugs that can inhibit PKM2 activity are needed for both clinic and research, and compound 6a may prove useful in this context.

#### 3.2.4. Predicted Toxicity and Pharmacokinetic Properties

A set of relevant properties of compound **6a** was calculated for the evaluation of chemical and biological properties and compared with controls used in the clinic (carboplatin and doxorubicin) from its 2D chemical structure using SwissADME and admetSAR 2 servers. The Lipinski “rule of 5” is used to evaluate oral bioavailability according to four parameters. The parameters are: (1) logarithm of the octanol/water partition coefficient (LogP < 5); (2) number of hydrogen bond acceptors (nON < 10); (3) number of hydrogen bond donors (nOH/NH < 5); and (4) molecular weight (MW < 500 Da) [46]. Compounds with two or more violations of these criteria probably do not have good permeation and absorption. Overall, compound **6a** fulfilled the Lipinski “rule of 5” with no violation to the rule, compared to controls, whereas doxorubicin scores three and carboplatin zero (Table 4).

Another parameter used to predict the quality of the new compound, the topological polar surface areas (TPSA), was estimated for **6a**, doxorubicin and carboplatin. TPSA is used in chemistry to model medicinal product absorption and optimize a drug ability to permeate cells. The TPSA of a compound is defined as the sum of the surface area of all polar atoms, which consist mainly of oxygen and nitrogen, as well as hydrogens [47]. Compounds with a TPSA greater than 140 Å have low permeability in the cell membrane, while molecules with PSA lower than 60 Å have high permeability and human intestinal absorption [48]. The TPSA of **6a** was 83.91 Å (Table 4), suggesting a moderate cell permeability that was better than doxorubicin (206.1 Å) and close to carboplatin (126.6 Å).

To reinforce the rule-based prediction of absorption and permeability, we also predicted the bioavailability of compound **6a** using a QSAR-based method available within admetSAR 2.0 server. The results showed compound **6a** with good oral bioavailability (Table 5). Doxorubicin and carboplatin on the other hand were predicted to have a poor oral bioavailability. Indeed, experimental studies have demonstrated the low oral bioavailability of these drugs [49,50], proving the reliability of our predictions, which in turn supports that compound **6a** is more likely for oral delivery, unlike the anticancer drugs evaluated. Taken together, these analyses suggest a good oral bioavailability of **6a** in comparison to controls.

Furthermore, one of the challenges of chemotherapy is the multiple drug resistance (MDR) phenotype of patients to chemotherapy leading to treatment failure and cancer relapse. The phosphoglycoprotein (P-gp) enzymes family, one of the main MDR effectors are an efflux transporter protein family that constitute a mechanism of resistance to drugs [51]. Compound **6a** was predicted to be an inhibitor of P-gp multidrug transporter, but not a substrate, in opposition to doxorubicin. Additionally, carboplatin was not predicted as substrate nor inhibitor of this P-gp (Table 5). These predictions are in agreement with the experimental data available for both control drugs [49]. All the above in silico analyses suggest that compound **6a**, in addition to having a good pharmacological profile, might be orally absorbed, could also act as a P-gp inhibitor, and is not substrate of P-gp, increasing its likelihood as a good drug lead.

#### 3.2.5. Investigation of the Cell Death Pathway

After defining that the compound **6a** is selective and tolerated in animals (LD_50_: 150 mg/kg), that the molecule is a possible ligand to topoisomerases and PKM2 enzymes and that it presents a desirable pharmacological profile, we next aimed to determine the cell death pathway induced by this compound. Chemotherapy plays an important role in inducing different types of cell death, such as apoptosis, necrosis/necroptosis, and autophagy. Hence, the determination of cell death pathways is essential for the development of new antitumor drugs [52].

**Figure 3:**
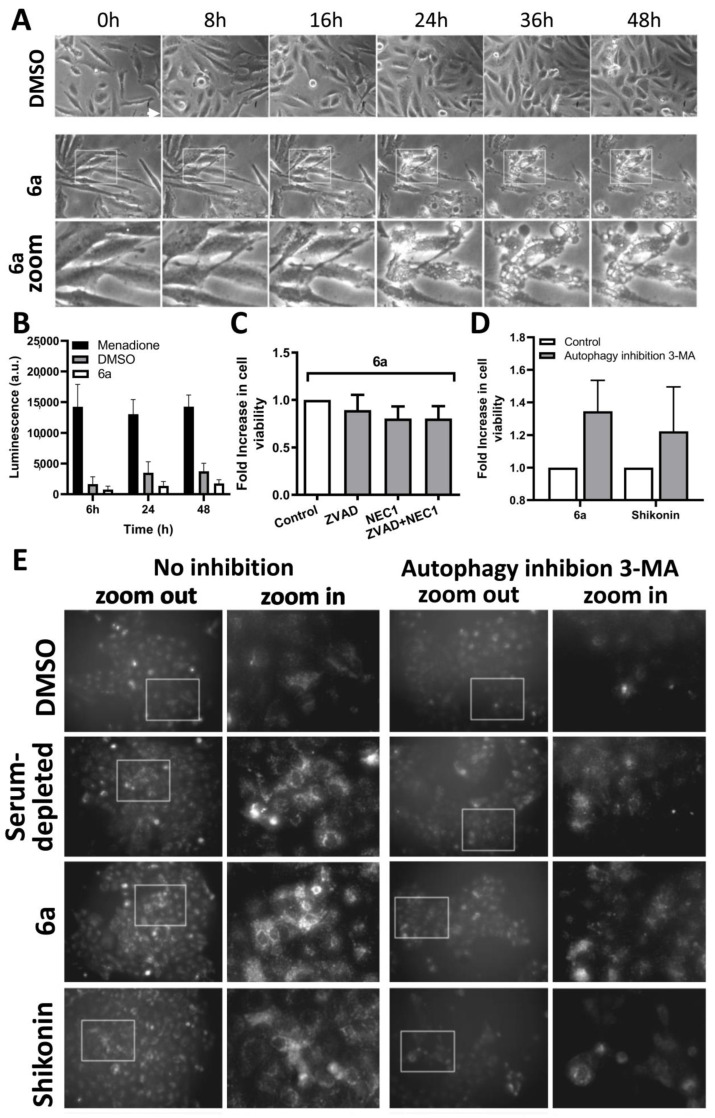
Compound **6a** induces cell death by autophagy and late apoptosis. All experiments were done using SCC9 cells. (**A**) Time-lapse video microscopy images obtained from Supplementary Video S1 reveal that **6a** (2 × IC_50_) induces the appearance of bubbles and loss of membrane integrity, swelling of the vesicles inside the cells and cellular rupture, which were intensified at 36 and 48 h. The same result was not observed for the DMSO control. (**B**) Reactive oxygen species (ROS) analysis with cell treated with compound **6a** for 6, 24 and 48 h using DMSO and menadione as controls. (**C**) Cytotoxicity assay by MTT in SCC-9 cells using pan-caspase inhibitor (ZVAD) and necrostatin necroptosis inhibitor with cell treated with 1 × IC_50_ of the compound **6a** for 24 h. (**D**) MTT assay in SCC9 cells using autophagy inhibitor 3-MA in cells treated with 1 × IC_50_ of compound **6a**, shikonin and DMSO were used as controls. (**E**) Autophagosome labeling. Using the autophagy assay kit (material and methods) in cells treated with 1 × IC_50_ of the compound **6a**, shikonin, serum depleted medium (positive control) and DMSO (negative control). Cells were photographed on a Zeiss inverted fluorescence microscope. Result of at least 3 independent experiments.

Apoptosis is a process of programmed cell death that occurs through a sequence of molecular events, such as cells shrinkage, DNA fragmentation and cell releases of membrane-enclosed contents called apoptotic bodies [53]. We used time-lapse video microscopy images (Figure 3A and Supplementary Video S1) to analyze whether the morphological characteristics would indicate cell death morphology by apoptosis. We verified that the cells treated with **6a** did not suffer shrinkage and did not present small membrane blebs throughout the analyzed time (Figure 3A). On the other hand, after 24 h of treatment, membrane bubbles appeared and grew progressively, followed by an increase in the number and size of vesicles inside the cells with subsequent membrane permeabilization, which were not observed in the DMSO control (Figure 3A). One of the possible mechanisms of death induction that leads to morphological phenotypes similar to that observed is the production and accumulation of ROS in cells. Different naphthoquinones are capable of inducing production of ROS, being one of the properties that most confer antineoplastic characteristics to this class of substances [36,54,55]. The oxidative activity of compound **6a** was compared with another naphthoquinone already shown to be a major producer of ROS, menadione. There was a low production of ROS in SCC9 cells treated with compound **6a**, close to that observed for DMSO and inferior to the positive control menadione, showing that compound **6a** does not trigger ROS production (Figure 3B).

In order to better investigate the process of cell death, we performed assays with the pan-caspase inhibitor ZVAD and with the necroptosis inhibitor necrostatin, or in combination, in which we observed no significant difference in the percentage of cell viability between the different conditions (Figure 3C), indicating that possibly neither apoptosis nor necroptosis is the cell death mechanism involved in **6a** cytotoxicity. Considering the result of the video microscopy images (Figure 3A and Supplementary video), the vesicles present inside cells could be an indicative of cell death induced by autophagy. In fact, studies show that some naphthoquinones have the potential to induce cell death by autophagy, with shikonin [56,57], plumbagin [58,59], and alkannin [60] as examples. As seen in Figure 3D, the autophagy inhibitor 3-MA [61] reduced cell death induced by **6a** and the control shikonin. To confirm the autophagic phenotype, we performed the labeling of autophagosomes where it was possible to visualize the small vesicles formed after 36 h of treatment with **6a**, demonstrating the greater presence of vesicles compared to the positive control of serum-deprivation and similarly to shikonin (Figure 3E). Moreover, this phenotype was completely reversed by treatment with autophagy inhibitor (3-MA), strongly suggesting that the observed death occurs by autophagy.

Pyruvate kinase M2 (PKM2) is an enzyme that plays a key role in tumor progression, both in gene transcription and in the production of ATP and intermediates of the glycolytic pathway, through the Warburg effect [41,62]. Through molecular docking, we proposed that **6a** possibly inhibits PKM2 with a stronger binding affinity than that observed for already known inhibitors, such as lapachol and shikonin. In OSCC, PKM2 expression is associated with cell growth, invasion, and tumor progression [63,64,65]. Therefore, it is possible to correlate the results of molecular docking with cell death assays. Compound **6a**, when inhibiting PKM2, possibly leads the tumor cell to energy deprivation, entering the autophagic process in search of building blocks and ATP.

**Figure 4:**
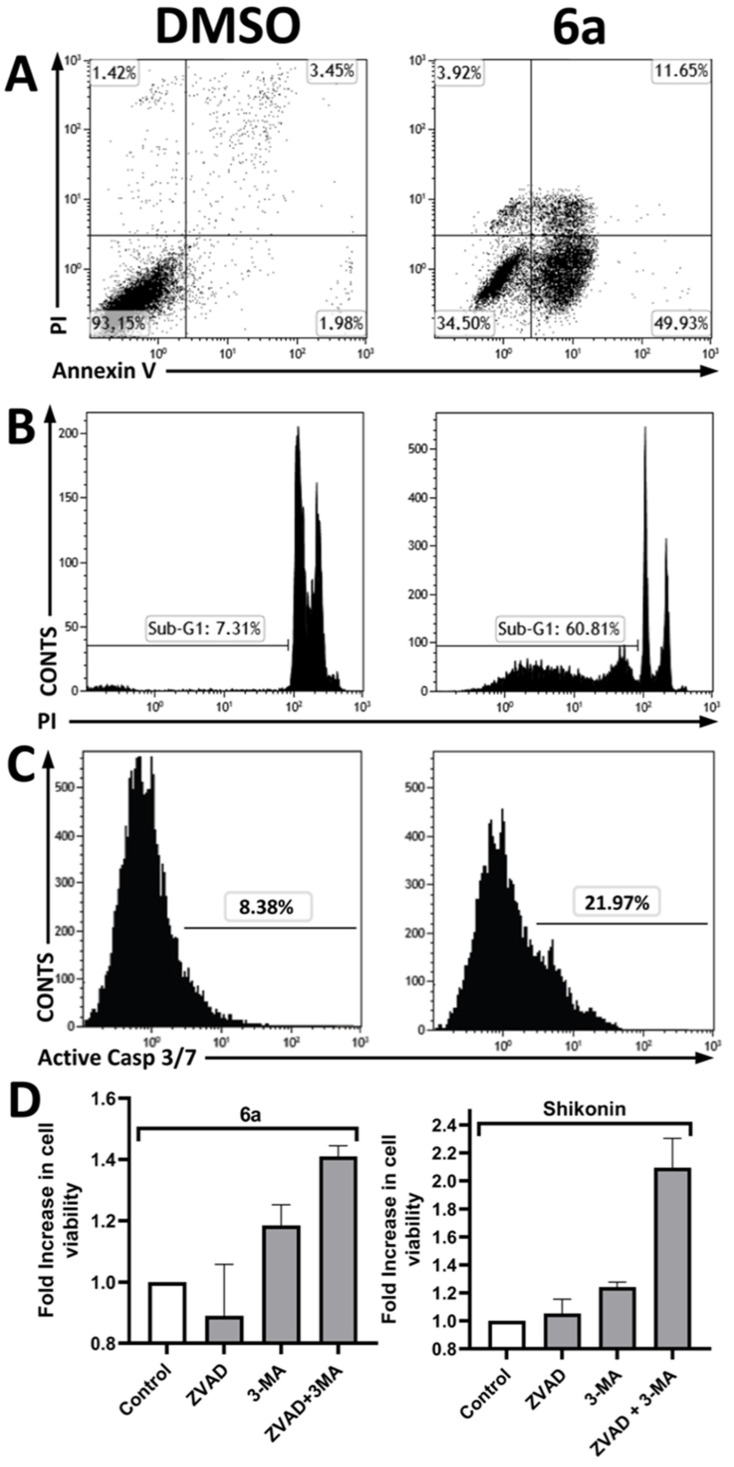
Compound **6a** induces a late apoptotic phenotype possibly induced by autophagy. All experiments used SCC9 cells. (**A**) Analysis of phosphatidylserine exposure 48 h after plating. Cells were stained with annexin-V conjugated to FITC and P.I. and analyzed by flow cytometry. (**B**) Analysis of cell death by propidium iodide (PI) staining. After 48 h of treatment, cells were trypsinized and stained with propidium iodide in the presence of NP-40. The percentage of cell death (sub-G_0_ DNA content) is indicated. (**C**) Flow cytometry data representative of caspase 3/7 activity. SCC9 cells were treated with the equivalent of 2 × IC_50_ of compound **6a** and DMSO was used as a control for 48 h of treatment. The activity of caspase 3/7 of the cells increased significantly under **6a** treatment compared to the control. (**A**–**C**) Results are representative of data from at least three independent experiments. (**D**) MTT assay of cells treated with 1 × IC_50_ of compound **6a**, shikonin or DMSO for 24 h. Autophagy inhibitor (3-MA) and pan-caspase inhibitor (ZVAD), alone or in combination, were used to analyze cell viability. Data were represented as mean SD from three independent experiments.

Although autophagy is a process associated with cell survival, when it occurs in excess and for long periods, it can trigger late cell death by apoptosis or necroptosis [66,67]. We subsequently investigated these late cell death pathways by flow cytometry. Through these analyses, we observed that the process of autophagy resulting from the treatment with **6a** may cause a late apoptosis process (only after 48 h and which is not reversible with ZVAD alone). Treatment of SCC9 cells with **6a** led to a ~50% stain for single positive phosphatidyl serin exposition (Figure 4A), DNA fragmentation in ~60% of the cells (Figure 4B, Sub-G_1_ DNA-content), and activated effector caspase 3/7 labeling (Figure 4C) in ~22%, all indicative of death by apoptosis. The results in Figure 4D reinforce these results, where treatment with ZVAD alone led to no change in the cell viability in contrast to treatment with 3-MA where there is an increase in cell viability compared to treatment with **6a** alone. However, when ZVAD is associated with 3-MA, there is a greater protection from the death process. The same result was observed for shikonin, which indicates that these naphthoquinones can act similarly in OSCC cells, but **6a** is 2.4 times more selective than shikonin. It is noteworthy that **6a** does not induce any significant cell cycle alteration in SCC9 cells (Supplementary Figure S2) and induces a low level of PI positive cells indicative of necrosis (Figure 4A).

Recent studies showed that PKM2 knockout inhibits the AKT/mTOR signaling pathway in cancer cells by suppressing protein expression important in glycogen synthesis, which contributes to the process of cell death by autophagy [68]. These results are related to those seen for naphthoquinone plumbagin, a PKM2 inhibitor, which is capable of inducing cell death by autophagy followed by apoptosis in SCC via the p38 MAPK and PI3K/Akt/mTOR pathways [69], for shikonin, which inhibits PKM2 and other glycolytic enzymes, inhibiting intracellular ATP levels through PI3K/Akt/mTOR inhibition pathways [69], and for shikonin, which inhibits PKM2 and other glycolytic enzymes, inhibiting intracellular ATP levels through PI3K/Akt/mTOR inhibition [61]. The results obtained for **6a** indicate that this is a promising compound for the treatment of OSCC, being capable to act as an inhibitor of enzymes essential for tumor cell metabolism, leading to cell death by autophagy and apoptosis, being selective, tolerable in animals, and with good pharmacokinetic characteristics.

## 4. Conclusions

In summary, sixteen new Mannich adducts from naphthoquinones were synthesized and evaluated for their antitumor potential in OSCC cells. Compound **6a** was very cytotoxic (56 µM) and selective (SI 2.36) among those tested and at levels comparable and generally superior to chemotherapeutic controls; and is slightly toxic at concentrations below 150 mg/kg in animals, but well tolerated at 100 mg/kg. Compound **6a** was shown to have a pharmacokinetic profile within desirable parameters for drug development. This compound is possibly able to bind to enzymes important for tumor progression, such as PKM2 and topoisomerases in allosteric inhibition sites. Our data show that this compound can induce cell death by autophagy followed by late apoptosis in the OSCC, showing promise for future preclinical trials.

## Figures and Tables

**Table 1:** Determination of IC_50_ and selectivity index of Mannich adducts from naphthoquinones. SCC-9 (OSCC cells) and normal human gingival fibroblasts were treated with the indicated compounds for 48 h and cell viability was determined as indicated in materials and methods. Shown from left to right: compound nomenclature, SCC-9 tumor or normal fibroblast, IC_50_ (μM), SD and selectivity index (IS) from at least 3 independent experiments. IS = IC_50_ of the compound in cells of normal oral fibroblasts/IC_50_ of the same compound for oral cancer lineage SCC-9. SD = standard deviation. Most selective compound were gray shaded.

Compounds	SCC9—Oral Cancer		Primary Gengival Fibroblast		Selective Index (S.I.)
	IC_50_ (µM)	SD	IC_50_ (µM)	SD	
**5a**	23.2	0.05	37.5	0.09	1.61
**5b**	28.5	0.04	55.7	0.04	1.95
**5c**	24.8	0.07	38.3	0.09	1.54
**5d**	38.8	0.02	69.1	0.01	1.78
**5e**	22.1	0.07	40.1	0.05	1.81
**5f**	22.2	0.06	45.7	0.07	2.05
**5g**	21.0	0.08	35.5	0.05	1.68
**5h**	24.9	0.12	49.1	0.05	1.97
**5i**	23.9	0.05	42.6	0.09	1.77
**6a**	56.2	0.07	186.6	0.07	3.31
**6b**	82.2	0.02	229.8	0.06	2.79
**6c**	56.1	0.09	148.1	0.05	2.63
**6d**	73.0	0.03	155.1	0.04	2.12
**6e**	73.2	0.06	196.7	0.06	2.68
**6h**	11.1	0.17	53.7	0.08	4.83
**6i**	70.7	0.14	73.5	0.14	1.03
Doxorrubicin	7.2	0.04	18.68	0.40	2.57
Carboplatin	223.0	0.04	192.8	0.03	0.85
Lapachol	42.1	0.08	101.6	0.09	2.41
Shikonin	1.8	0.02	1.72	0.05	0.95

**Table 2:** Characterization of the most selective compounds in other OSCC cells. The IC_50_ (μM) of three different OSCC cell lines (SCC 4, SCC-9 and SCC-25) and normal fibroblast cells followed by the average of the selectivity index of compounds **6a** and **6h** were calculated in the same way as in Table 1. All experiments are results of at least 3 independent experiments.

	Oral Tumor Cells							Primary Gingival Fibroblast		Average S.I.
Compound	SCC9		SCC25		SCC4		Mean			
	IC_50_	S.D.	IC_50_	S.D.	IC_50_	S.D.		IC_50_	S.D.	
**6a**	56.24	0.07	87.28	0.04	92.81	0.06	78.70	186.60	0.07	2.36
**6h**	11.10	0.17	56.60	0.05	98.39	0.03	44.58	53.73	0.08	1.29
Carboplatin	223.00	0.04	264.00	0.02	195.00	0.06	227.50	192.80	0.03	0.85
Doxorubicin	7.20	0.04	1.12	0.06	5.87	0.03	4.73	18.68	0.40	3.94
Lapachol	42.12	0.08	85.85	0.07	55.32	0.05	61.09	101.60	0.09	1.66
Shikonin	1.81	0.02	1.50	0.05	1.91	0.05	1.74	1.72	0.05	0.98

**Table 3:** Acute toxicity study: morbidity, mortality, gross organs necropsy and histology of mice treated with compound **6a**. For more information and results, see Supplemental Table S1.

Treatment	Dose mg/kg ^a^	Change in Body Weight	Change in Food Consumption	Morbidity ^b^	Mortality	Gross Necropsy ^c^	Histology ^d^
Control	0 *n* = 3	Absent	Absent	Absent	Absent	No alteration	Normal
**6a**	100 *n* = 3	Absent	Absent	Absent	Absent	No alteration	Mild kidney and portal hyperemia and mild liver intracytoplasmic degeneration
	150 *n* = 4	Absent	ND ^e^	Low activity and back-arching	50% of death	Precipitaded compound in loco	Mild arterial and venous hyperemia at the lung, kidney and liver portal and liver intracytoplasmic degeneration
	200 *n* = 3	ND ^e^	ND ^e^	Low activity and back-arching	100% of death	Precipitaded compound in loco	Mild arterial and venous hyperemia at thelung, kidney and liver portal and liver intracytoplasmic degeneration at time of death

^a^ Dose level for each subsequent group was determined based on the result after the previous lower dosing. *n* stands for the number of animals used in each experiment. ^b^ Morbidity symptoms were analyzed every day, two times a day and were considered as: tremors, convulsion, salivation, diarrhea, lethargy, coma, signs of pain, and mobility defect. ^c^ Gross organ necropsy of liver, thymus, right kidney, right testicle, heart, major lymph nodes and lung. ^d^ Histopathology of the lung, kidney, heart, liver and spleen was accessed by trained pathologist and are summarized here. ^e^ Not detected.

**Table 4:** Physico-chemical descriptors related to pharmacokinetic properties **6a** in comparison to the chemotherapy drugs doxorubicin and carboplatin.

Compounds	cLogP	nON	nOH/NH	MW	Lipinski’s Violations ^a^	TPSA (Å^2^)
**6a**	2.54	5	1	461	0	83.91
Doxorubicin	−2.10	12	6	543	3	206.1
Carboplatin	−1.79	6	4	371	0	126.6

^a^ Number of violations to the Lipinski “rule of five”: logP < 5; MW, molecular weight < 500; nON, number of hydrogen bond acceptors < 10; and nOH/NH, number of hydrogen bond donors < 5.

**Table 5:** Properties analyzed in admetSAR. The properties analyzed were oral bioavailability, substrate and P-gp inhibitor. The analysis was performed with compound **6a** and the chemotherapeutic agents, carboplatin and doxorubicin as controls.

ADMET	6a	Carboplatin	Doxorrubicin
Oral Bioavailability	+0.63	−0.60	−0.91
P-glycoprotein inhibitor	+0.82	−0.99	−0.92
P-glycoprotein substrate	−0.56	−0.99	+0.95

## Data Availability

Not applicable.

## Supplementary Materials

The following supporting information can be downloaded at: https://www.mdpi.com/article/10.3390/molecules28010309/s1, Figure S1: Acute toxicity study shows the mean body weight variation (A) and food consumption (B); Figure S2: Differences in cell cycle distribution. Cell cycle distribution was analyzed after propidium iodide staining and FACS analyses. SCC9 lineage cells were plated in a 6-well plate (5 × 105 cells/well). The phases (sub G1), G0/G1, S and G2 of the cell cyclewere classified based on the DNA content after staining with iodide of propidium (PI). Each image is representative of at least three independent experiments; Table S1: Average histopathological findings of 3 animals group (4 at 150 mg/kg) treated with indicated compound and concentration; Video S1: video microscopy images; NMR spectral images, IR spectral images, and Hight resolution mass spectral (HRMS) of the compounds **5** and **6**.

## Legal Aspects

The use of animals was authorized by the Ethics Committee on Animal Use of the Universidade Federal Fluminense with registration number 982. The use of human blood was approved by the Research Ethics Committee of the Fluminense Federal University—Nova Friburgo, RJ (CAAE: 43134721.4.0000.5626).

## References

[1] Chi A.C., Day T.A., Neville B.W. (2015). Oral cavity and oropharyngeal squamous cell carcinoma-an update. CA Cancer J. Clin..

[2] Siegel R.L., Miller K.D., Jemal A. (2019). Cancer statistics, 2019. CA Cancer J. Clin..

[3] Speight P.M., Farthing P.M. (2018). The pathology of oral cancer. Br. Dent. J..

[4] Chai A.W.Y., Lim K.P., Cheong S.C. (2020). Translational genomics and recent advances in oral squamous cell carcinoma. Semin. Cancer Biol..

[5] Li C.C., Shen Z., Bavarian R., Yang F., Bhattacharya A. (2020). Oral Cancer: Genetics and the Role of Precision Medicine. Surg. Oncol. Clin. N. Am..

[6] Güneri P., Epstein J.B. (2014). Late stage diagnosis of oral cancer: Components and possible solutions. Oral Oncol..

[7] Aminin D., Polonik S. (2020). 1,4-Naphthoquinones: Some biological properties and application. Chem. Pharm. Bull..

[8] Pereyra C.E., Dantas R.F., Ferreira S.B., Gomes L.P., Silva F.P. (2019). The diverse mechanisms and anticancer potential of naphthoquinones. Cancer Cell Int..

[9] Biersack B., Ahmed K., Padhye S., Schobert R. (2018). Recent developments concerning the application of the Mannich reaction for drug design. Expert Opin. Drug Discov..

[10] Roman G. (2015). Mannich bases in medicinal chemistry and drug design. Eur. J. Med. Chem..

[11] Machado T.Q., Felisberto J.R.S., Guimarães E.F., de Queiroz G.A., da Fonseca A.C.C., Ramos Y.J., Marques A.M., Moreira D.L., Robbs B.K. (2021). Apoptotic effect of β-pinene on oral squamous cell carcinoma as one of the major compounds from essential oil of medicinal plant *Piper rivinoides* Kunth. Nat. Prod. Res..

[12] Macedo A.L., da Silva D.P.D., Moreira D.L., de Queiroz L.N., Vasconcelos T.R.A., Araujo G.F., Kaplan M.A.C., Pereira S.S.C., de Almeida E.C.P., Valverde A.L. (2019). Cytotoxicity and selectiveness of Brazilian Piper species towards oral carcinoma cells. Biomed. Pharmacother..

[13] Parasuraman S. (2011). Toxicological screening. J. Pharmacol. Pharmacother..

[14] Lucena P.I., Faget D.V., Pachulec E., Robaina M.C., Klumb C.E., Robbs B.K., Viola J.P.B. (2016). NFAT2 Isoforms Differentially Regulate Gene Expression, Cell Death, and Transformation through Alternative N-Terminal Domains. Mol. Cell. Biol..

[15] Faget D.V., Lucena P.I., Robbs B.K., Viola J.P.B. (2012). NFAT1 C-Terminal Domains Are Necessary but Not Sufficient for Inducing Cell Death. PLoS ONE.

[16] Trott O., Olson A.J. (2009). AutoDock Vina: Improving the speed and accuracy of docking with a new scoring function, efficient optimization, and multithreading. J. Comput. Chem..

[17] Costa D.C.S., de Almeida G.S., Rabelo V.W.-H., Cabral L.M., Sathler P.C., Abreu P.A., Ferreira V.F., da Silva L.C.R.P., da Silva F.C. (2018). Synthesis and evaluation of the cytotoxic activity of Furanaphthoquinones tethered to 1H-1,2,3-triazoles in Caco-2, Calu-3, MDA-MB231 cells. Eur. J. Med. Chem..

[18] Zorzanelli B.C., Ouverney G., Pauli F.P., da Fonseca A.C.C., de Almeida E.C.P., de Carvalho D.G., Possik P.A., Rabelo V.W.-H., Abreu P.A., Pontes B. (2022). Pro-Apoptotic Antitumoral Effect of Novel Acridine-Core Naphthoquinone Compounds against Oral Squamous Cell Carcinoma. Molecules.

[19] Neves A.P., Barbosa C.C., Greco S.J., Vargas M.D., Visentin L.C., Pinheiro C.B., Mangrich A.S., Barbosa J.P., da Costa G.L. (2009). Novel Aminonaphthoquinone Mannich Bases Derived from Lawsone and their Copper(II) Complexes: Synthesis, Characterization and Antibacterial Activity. J. Braz. Chem. Soc..

[20] da Rocha D.R., de Souza A.C., Resende J.A., Santos W.C., dos Santos E.A., Pessoa C., de Moraes M.O., Costa-Lotufo L.V., Montenegro R.C., Ferreira V.F. (2011). Synthesis of new 9-hydroxy-α- and 7-hydroxy-β-pyran naphthoquinones and cytotoxicity against cancer cell lines. Org. Biomol. Chem..

[21] Ribeiro R.C.B., de Freitas P.P., Moreira C.S., de Moraes L.G.C., de Moraes M.G., da Silva F.C., Rocha D.R., Gimba E.R.P., Ferreira V.F. (2020). A New Strategy for the Synthesis of Nonsymmetrical 3,3′-(Aryl/alkyl- methylene) bis-2-hydroxy-1,4-naphthoquinones and Their Cytotoxic Effects in PC3 Prostate Cancer Cells. J. Braz. Chem. Soc..

[22] Hartner L. (2018). Chemotherapy for Oral Cancer. Dent. Clin. N. Am..

[23] Ho G.Y., Woodward N., Coward J.I.G. (2016). Cisplatin versus carboplatin: Comparative review of therapeutic management in solid malignancies. Crit. Rev. Oncol. Hematol..

[24] Khasraw M., Bell R., Dang C. (2012). Epirubicin: Is it like doxorubicin in breast cancer? A clinical review. Breast.

[25] Shafei A., El-Bakly W., Sobhy A., Wagdy O., Reda A., Aboelenin O., Marzouk A., El Habak K., Mostafa R., Ali M.A. (2017). A review on the efficacy and toxicity of different doxorubicin nanoparticles for targeted therapy in metastatic breast cancer. Biomed. Pharmacother..

[26] Boulos J.C., Rahama M., Hegazy M.-E.F., Efferth T. (2019). Shikonin derivatives for cancer prevention and therapy. Cancer Lett..

[27] Epifano F., Genovese S., Fiorito S., Mathieu V., Kiss R. (2014). Lapachol and its congeners as anticancer agents: A review. Phytochem. Rev..

[28] Chipoline I.C., da Fonseca A.C.C., da Costa G.R.M., de Souza M.P., Rabelo V.W.-H., de Queiroz L.N., de Souza T.L.F., de Almeida E.C.P., Abreu P.A., Pontes B. (2020). Molecular mechanism of action of new 1,4-naphthoquinones tethered to 1,2,3-1H-triazoles with cytotoxic and selective effect against oral squamous cell carcinoma. Bioorg. Chem..

[29] Zorzanelli B.C., de Queiroz L.N., Santos R.M., Menezes L.M., Gomes F.C., Ferreira V.F., da Silva F.C., Robbs B.K. (2018). Potential cytotoxic and selective effect of new benzo [b] xanthenes against oral squamous cell carcinoma. Fut. Med. Chem..

[30] Militello C., Rundo L., Minafra L., Cammarata F.P., Calvaruso M., Conti V., Russo G. (2020). MF2C3: Multi-Feature Fuzzy Clustering to Enhance Cell Colony Detection in Automated Clonogenic Assay Evaluation. Symmetry.

[31] Pereira V.S.S., de Oliveira C.B.S., Fumagalli F., Emery F.S., da Silva N.B., de Andrade-Neto V.F. (2016). Cytotoxicity, hemolysis and in vivo acute toxicity of 2-hydroxy-3-anilino-1,4-naphthoquinone derivatives. Toxicol. Rep..

[32] Ahmed M. (2015). Acute Toxicity (Lethal Dose 50 Calculation) of Herbal Drug Somina in Rats and Mice. Pharmacol. Pharm..

[33] Liu W., Chen X., Ye Q., Hou S., Lou L., Xie C. (2012). 3-Hydroxycarboplatin, a simple carboplatin derivative endowed with an improved toxicological profile. Platin. Met. Rev..

[34] Boonyalai N., Sittikul P., Pradidphol N., Kongkathip N. (2013). Biophysical and molecular docking studies of naphthoquinone derivatives on the ATPase domain of human Topoisomerase II. Biomed. Pharmacother..

[35] Delgado J.L., Hsieh C.-M., Chan N.-L., Hiasa H. (2017). Topoisomerases as Anticancer Targets. Biochem. J..

[36] da Silva M.N., Ferreira V.F., de Souza M.C.B.V. (2003). Um panorama atual da química e da farmacologia de naftoquinonas, com ênfase na beta-lapachona e derivados. Quím. Nova.

[37] Steves-Souza A., Figueiredo D.V., Esteves A., Câmara C.A., Vargas M.D., Pinto A.C., Echevarria A. (2007). Cytotoxic and DNA-topoisomerase effects of lapachol amine derivatives and interactions with DNA. Braz. J. Med. Biol. Res..

[38] Ferreira S.B., Gonzaga D.T.G., Santos W.C., Araújo K.G.L., Ferreira V.F. (2010). ß-Lapachone: Medicinal chemistry significance and structural modifications. Rev. Virtual Quím..

[39] Gurbani D., Kukshal V., Laubenthal J., Kumar A., Pandey A., Tripathi S., Arora A., Jain S.K., Ramachandran R., Anderson D. (2012). Mechanism of inhibition of the ATpase domain of human topoisomerase IIα by 1,4-benzoquinone, 1,2-naphthoquinone, 1,4-naphthoquinone, and 9,10-phenanthroquinone. Toxicol. Sci..

[40] Wellington K.W. (2015). Understanding cancer and the anticancer activities of naphthoquinones-a review. RSC Adv..

[41] Chhipa A.S., Patel S. (2021). Targeting pyruvate kinase muscle isoform 2 (PKM2) in cancer: What do we know so far?. Life Sci..

[42] Babu M.S., Mahanta S., Lakhter A.J., Hato T., Paul S., Naidu S.R. (2018). Lapachol inhibits glycolysis in cancer cells by targeting pyruvate kinase M2. PLoS ONE.

[43] Chen J., Xie J., Jiang Z., Wang B., Wang Y., Hu X. (2011). Shikonin and its analogs inhibit cancer cell glycolysis by targeting tumor pyruvate kinase-M2. Oncogene.

[44] Zhao X., Zhu Y., Hu J., Jiang L., Li L., Jia S., Zen K. (2018). Shikonin Inhibits Tumor Growth in Mice by Suppressing Pyruvate Kinase M2-mediated Aerobic Glycolysis. Sci. Rep..

[45] Larsen T.M., Benning M.M., Rayment I., Reed G.H. (1998). Structure of the Bis(Mg^2+^)-ATP-oxalate complex of the rabbit muscle pyruvate kinase at 2.1 Å resolution: ATP binding over a barrel. Biochemistry.

[46] Lipinski C.A., Lombardo F., Dominy B.W., Feeney P.J. (2001). Experimental and computational approaches to estimate solubility and permeability in drug discovery and development settings. Adv. Drug Deliv. Rev..

[47] Cardoso M.F.D.C., Salomão K., Bombaça A.C., da Rocha D.R., da Silva F.C., Cavaleiro J.A.S., de Castro S.L., Ferreira V.F. (2015). Synthesis and anti-Trypanosoma cruzi activity of new 3-phenylthio-nor-β-lapachone derivatives. Bioorg. Med. Chem..

[48] Palm K., Stenberg P., Luthman K., Artursson P. (1997). Polar Molecular Surface Properties Predict the Intestinal Absorption of Drugs in Humans. Pharm. Res..

[49] Alrushaid S., Sayre C.L., Yáñez J.A., Forrest M.L., Senadheera S.N., Burczynski F.J., Löbenberg R., Davies N.M. (2017). Pharmacokinetic and Toxicodynamic Characterization of a Novel Doxorubicin Derivative. Pharmaceutics.

[50] Oguri S., Sakakibara T., Mase H., Shimizu T., Ishikawa K., Kimura K., Smyth R.D. (1988). Clinical Pharmacokinetics of Carboplatin. Clin. Pharmacol..

[51] Huber P.C., Maruiama C.H., Almeida W.P. (2010). Glicoproteína-P, resistência a múltiplas drogas (MDR) e relação estrutura-atividade de moduladores. Quím. Nova.

[52] Mansilla S., Llovera L., Portugal J. (2012). Chemotherapeutic Targeting of Cell Death Pathways. Anticancer Agents Med. Chem..

[53] Xu X., Lai Y., Hua Z.C. (2019). Apoptosis and apoptotic body: Disease message and therapeutic target potentials. Biosci. Rep..

[54] Jamier V., Ba L.A., Jacob C. (2010). Selenium- and tellurium-containing multifunctional redox agents as biochemical redox modulators with selective cytotoxicity. Chem. Eur. J..

[55] Li K., Wang B., Zheng L., Yang K., Li Y., Hu M., He D. (2018). Target ROS to induce apoptosis and cell cycle arrest by 5,7-dimethoxy-1,4-naphthoquinone derivative. Bioorg. Med. Chem. Lett..

[56] Gong K., Zhang Z., Chen Y., Shu H.-B., Li W. (2014). Extracellular signal-regulated kinase, receptor interacting protein, and reactive oxygen species regulate shikonin-induced autophagy in human hepatocellular carcinoma. Eur. J. Pharmacol..

[57] Shi S., Cao H. (2014). Shikonin promotes autophagy in BXPC-3 human pancreatic cancer cells through the PI3K/Akt signaling pathway. Oncol. Lett..

[58] Lin Y., Chen Y., Wang S., Ma J., Peng Y., Yuan X., Lv B., Chen W., Wei Y. (2019). Plumbagin induces autophagy and apoptosis of SMMC-7721 cells in vitro and in vivo. J. Cell. Biochem..

[59] Ma X., Yin X., Liu H., Chen Q., Feng Y., Ma X., Liu W. (2019). Antiproliferative activity of plumbagin (5-hydroxy-2-methyl-1,4-naphthoquinone) in human gastric carcinoma cells is facilitated via activation of autophagic pathway, mitochondrialmediated programmed cell death and inhibition of cell migration and invasion. J. BUON.

[60] Zheng Q., Li Q., Zhao G., Zhang J., Yuan H., Gong D., Guo Y., Liu X., Li K., Lin P. (2020). Alkannin induces cytotoxic autophagy and apoptosis by promoting ROS-mediated mitochondrial dysfunction and activation of JNK pathway. Biochem. Pharmacol..

[61] Li J., Pang J., Liu Z., Ge X., Zhen Y., Jiang C.C., Liu Y., Huo Q., Sun Y., Liu H. (2021). Shikonin induces programmed death of fibroblast synovial cells in rheumatoid arthritis by inhibiting energy pathways. Sci. Rep..

[62] Azoitei N., Becher A., Steinestel K., Rouhi A., Diepold K., Genze F., Simmet T., Seufferlein T. (2016). PKM2 promotes tumor angiogenesis by regulating HIF-1aα through NF-κB activation. Mol. Cancer.

[63] Kurihara-Shimomura M., Sasahira T., Nakashima C., Kuniyasu H., Shimomura H., Kirita T. (2018). The multifarious functions of pyruvate kinase M2 in oral cancer cells. Int. J. Mol. Sci..

[64] Kurihara-Shimomura M., Sasahira T., Shimomura H., Kirita T. (2020). Peroxidan plays a tumor-promoting role in oral squamous cell carcinoma. Int. J. Mol. Sci..

[65] Tanaka F., Yoshimoto S., Okamura K., Ikebe T., Hashimoto S. (2018). Nuclear PKM2 promotes the progression of oral squamous cell carcinoma by inducing EMT and post-translationally repressing TGIF2. Oncotarget.

[66] Denton D., Kumar S. (2019). Autophagy-dependent cell death. Cell Death Differ..

[67] Ryter S.W., Mizumura K., Choi A.M.K. (2014). The impact of autophagy on cell death modalities. Int. J. Cell Biol..

[68] Dey P., Kundu A., Sachan R., Park J.H., Ahn M.Y., Yoon K., Lee J., Kim N.D., Kim I.S., Lee B.M. (2019). PKM2 knockdown induces autophagic cell death via AKT/mTOR pathway in human prostate cancer cells. Cell. Physiol. Biochem..

[69] Pa S.T., Qin Y., Zhou Z.-W., He Z.-X., Zhang X., Yang T., Yang Y.-X., Wang D., Qiu J.-X., Zhou S.-F. (2015). Plumbagin induces G2/M arrest, apoptosis, and autophagy via p38 MAPK- and PI3K/Akt/mTOR-mediated pathways in human tongue squamous cell carcinoma cells. Drug Des. Devel. Ther..

