# Supplementary material for: Chemoselective Synthesis of Mannich Adducts from 1,4-Naphthoquinones and Profile as Autophagic Inducers in Oral Squamous Cell Carcinoma
Source: Molecules. 2022 Dec 30;28(1):309. doi: 10.3390/molecules28010309 (PMC9822194; doi:10.3390/molecules28010309)
Supplement: Supplementary file 1 [file molecules-28-00309-s001.zip › [Supplementary figures and table]-ForeziLSM SI (biological assays).pdf]

Supplementary materials

Chemoselective Synthesis of Mannich Adducts from 1,4-Naphthoquinones and Profile as Autophagic Inducers in Oral Squamous Cell Carcinoma

Amanda A. Borges [1,†], Michele P. de Souza [2,†], Anna Carolina C. da Fonseca [3], Guilherme F. Wermelinger [4], Ruan C. B. Ribeiro [1], Adriane A. P. Amaral [1], Cláudio José C. de Carvalho [1], Lucas S. Abreu [1], Lucas Nicolau de Queiroz [2], Elan C. P. de Almeida [4], Vitor W. Rabelo [5], Paula A. Abreu [5], Bruno Pontes [6], Vitor F. Ferreira [7], Fernando de C. da Silva [1], Luana da S. M. Forezi [1,*] and Bruno K. Robbs [1,*]

[1] Departamento de Química Orgânica, Instituto de Química, Campus do Valonguinho, Universidade Federal Fluminense, Niterói CEP 24020-150, Brazil
[2] Programa de Pós-Graduação em Ciências Aplicadas a Produtos para Saúde, Faculdade de Farmácia, Universidade Federal Fluminense, Niterói, CEP 24241-000, Brazil
[3] Programa de Pós-graduação em Odontologia, Instituto de Saúde de Nova Friburgo, Universidade Federal Fluminense, Nova Friburgo CEP 28625-650, Brazil.
[4] Departamento de Ciência Básica, Campus Universitário de Nova Friburgo, Universidade Federal Fluminense, Nova Friburgo CEP 28625-650, Brazil
[5] Instituto de Biodiversidade e Sustentabilidade, Campus Macaé, Universidade Federal do Rio de Janeiro, Macaé CEP 27965-045, Brazil
[6] Instituto de Ciências Biomédicas, Universidade Federal do Rio de Janeiro, Rio de Janeiro CEP 21941-902, Brazil
[7] Departamento de Tecnologia Farmacêutica, Faculdade de Farmácia, Universidade Federal Fluminense, Niterói CEP 24241-000, Brazil
* Correspondence: luanaforezi@id.uff.br (L.d.S.M.F.); brunokr@id.uff.br (B.K.R.)
† These authors contributed equally to this work.

Biological Assays

Supplementary Results and Methods:

Molecular modeling studies

A reverse docking approach was employed to evaluate the potential targets of compound **6a**. Considering that similar ligands bind to similar targets, a pool of six proteins was generated because they are known anticancer targets of lapachol and other naphthoquinone derivatives. The three-dimensional structure of these proteins was obtained in the Protein Data Bank (PDB) under the following codes: DNA-binding domain of topoisomerase I (PDB code 1K4T), IIα (PDB code 5GWK), and IIβ (PDB code 3QX3), ATPase domain of topoisomerase IIα (PDB code 1ZXM), humanpyruvate kinase M2 (hPKM2, PDB code 3SRD).

The three-dimensional structures of the R and S enantiomers of **6a**, shikonin, and lapachol were constructed using the Spartan'10 software (Wavefunction Inc. Irvine, CA, USA). The structures were first submitted to a conformational analysis in vacuum using the MMFF force field. Then, the lowest-energy conformer was optimized using the semi-empirical method RM1, followed by an energy calculation using the density functional theory (DFT) method with B3LYP/6-31G* basis set.

Molecular docking studies were carried out using Autodock Tools 1.5.7 and Autodock Vina 1.1.2 [1]. Docking protocols for topoisomerases I, IIα, and IIβ and hPKM2 have been validated and reported previously by our group [2,3]. The search parameters were kept as default. The docked and crystalized binding poses of this inhibitor were superimposed and showed a RMSD value of 0.58 Å, proving the excellent prediction accuracy of the docking protocol for this protein.

The lowest-binding-energy pose of the ligands with each enzyme was selected for interaction analysis using Discovery Studio Visualizer 2019 (Dassault Systèmes BIOVIA, San Diego, 2019) and Pymol v. 1.2r2 (The PyMOL Molecular Graphics System, Version 1.2r2 Schrödinger, LLC).

***In silico* target fishing for 6a by reverse docking**

To get more insights into the mechanism of action for the cytotoxic effects of the compound **6a**, we explored whether this compound could bind theoretically to the anticancer targets described for lapachol and other naphthoquinone derivatives by molecular docking. Six proteins were included in this study: the DNA binding domain of topoisomerase I, topoisomerase IIα, and IIβ, as well as the ATPase domain of topoisomerase IIα, the human pyruvate kinase M2 (hPKM2) [4-9]. Since **6a** was assayed as a racemic mixture, we docked both *R* and *S* enantiomers. According to their binding modes, our results suggested that the enantiomer *R* contributes mostly to the cytotoxic activity; thereby, we focused our analysis on this enantiomer.

Previously, molecular docking studies with topoisomerase I indicated that lapachol intercalates between the base pairs +1C/+1G and -1T/-1A like the inhibitor

topotecan, while its aliphatic chain bound towards the DNA minor groove [3]. Likewise, the compound **6a** bound in between the same base pairs as lapachol and topotecan, suggesting that it can inhibit this enzyme (Figure 1). In fact, the naphthoquinone ring of **6a** was superimposed with the rings A and B of topotecan and was π-stacked with +1G, +1C, and -1A. Unlike topotecan and lapachol, **6a** explores several amino acid residues of the enzyme, which may help to stabilize its binding mode. For instance, both the phenyl and chlorophenyl groups of **6a** were positioned towards the DNA major groove. The chlorine atom established a halogen bond with M428, whereas the ester oxygen was hydrogen-bonded with N352. Also, the phenyl ring was involved in van der Waals contacts with L429 and P431.

In case of topoisomerase IIβ, **6a** was stacked between the base pairs +1T/+4A and -1C/ +5G as lapachol and etoposide, a known inhibitor (Figure 1). The naphthoquinone ring of **6a** occupied the same site as the rings C and D of etoposide, and it was π-stacked with +1T and -1C bases and hydrogen-bonded with Q778. The ester oxygen interacted with the nucleobase -1C through a hydrogen bond as well. Additionally, the chlorophenyl and phenyl rings of **6a** bound towards the DNA major groove like the sugar moiety of etoposide, establishing a van der Waals contact with M782. Interestingly, this compound explored similar interactions as observed for lapachol and etoposide.

The anticancer activity of naphthoquinone derivatives can arise from the inhibition of targets other than topoisomerases. For instance, lapachol was shown to impair cancer cell metabolism by targeting the enzyme hPKM2 [4] as well as shikonin [10,11]. In our docking studies, both compounds occupied the entrance of the binding cavity, where the adenine moiety of ATP is expected to bind. Although **6a** was not superimposed to lapachol or shikonin, this compound still bound within this cavity and explored a deeper region where the phosphate groups of ATP bind in a homologous enzyme (Figure 1) [12]. Consequently, they exhibit a different interaction network. The chlorophenyl and phenyl rings of **6a** interacted with K207 and T328 via van der Waals interactions, respectively. Yet, we observed that the 1,4-naphthoquinone nucleus of the three compounds was involved in a hydrogen bond interaction with N75. The same group of **6a** was sandwiched between E118 and E272 through anion-π interactions, which probably makes its binding tighter and suggests this enzyme as a putative target for **6a** anticancer activity.

On the other hand, our results showed that **6a** could not intercalate between the DNA base pairs as observed for etoposide and lapachol in the DNA-binding domain of topoisomerase IIα. Likewise, this compound could not resemble the binding mode of known inhibitors of the ATPase domain of topoisomerase IIα. Therefore, the computational target fishing strategy used indicated that the **6a** could exert its anticancer action by inhibiting topoisomerase I and IIβ and hPKM2.

Acute toxicity:

Histopathology

Fragments of the lungs, heart, spleen, liver and kidneys of the animals of the different groups were collected. Fragments were fixed in 10% buffered formalin solution, dehydrated in decreasing dilutions of alcohol, diaphanized, and embedded in paraffin. The blocks were cut at 5 µm and the sections stained with routine staining Hematoxylin-Eosin (HE). The reading of the slides was performed in Nikon Eclipse E-200 microscope at different magnifications (40 X, 100 X, 200 X and 400 X) and the morphological alterations of each organ were duly observed and noted.

Table S1: Average histopathological findings of 3 animals group (4 at 150 mg/kg) treated with indicated compound and concentration. Legend: +: Discreet alteration; ++: Mild alteration; +++: High degree of alteration; N: No; Y: Yes; N/A: No alteration.

Organ	Histopathological findings	0 mg/kg			100 mg/kg			150 mg/kg				200 mg/kg		
		**Control**			**6a**			**6a**				**6a**		
Lung	Alveolar edema	N	N	N	N	N	N	N	+++	+++	N	N	++	+
	Arterial and venous hyperemia	+	+	+	+	+	+	++	+++	+++	++	+	++	+++
	Compensatory emphysema	+	+	+	+	+	+	+	+	+	+	+	+	+
	Obvious regional lymph node	N	N	N	N	Y	N	N	N	N	Y	N	N	N
Heart	Myocardium	N/A	N/A	N/A	N/A	N/A	N/A	N/A	N/A	N/A	N/A	N/A	N/A	N/A
	Valves	N/A	N/A	N/A	N/A	N/A	N/A	N/A	N/A	N/A	N/A	N/A	N/A	N/A
	Base Vessels	N/A	N/A	N/A	N/A	N/A	N/A	N/A	N/A	N/A	N/A	N/A	N/A	N/A
Kidney	Hyperemia	++	+	+	++	+	++	++	+	+	++	++	++	+
Liver	Centrolobular hyperemia	N	N	+	+	N	N	N	+	+	N	+	N	N
	Portal hyperemia	+	++	+	++	++	++	++	++	++	++	++	+	++
	Megalocytosis	+	+	+	+	+	+	+	+	+	+	+	+	+
	Intracytoplasmic vacuolar degeneration	+	+	+	++	++	++	++	++	+	++	++	N	+
	Obvious hepatocyte cords	N	N	N	N	N	N	N	N	N	N	N	N	N
	Perivascular and periportal lymphocyte focus	Y	N	N	N	Y	Y	N	N	Y	N	N	N	N
	Hepatocyte Binucleation	+	+	+	+	+	+	+	+	+	+	++	+	+
Spleem	Red pulp	N/A	+	N/A	N/A	N/A	+	+	N/A	N/A	N/A	N/A	N/A	N/A
	White pulp	N/A	N/A	N/A	N/A	N/A	N/A	N/A	N/A	N/A	N/A	N/A	N/A	N/A

A)

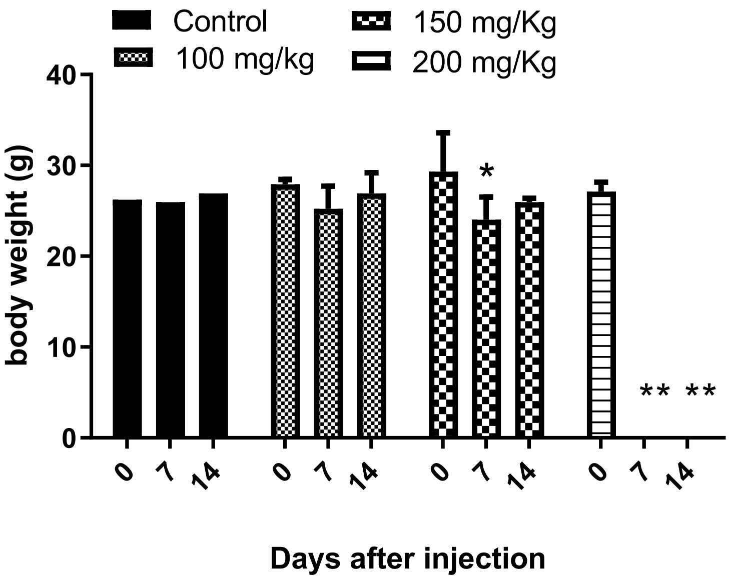

B)

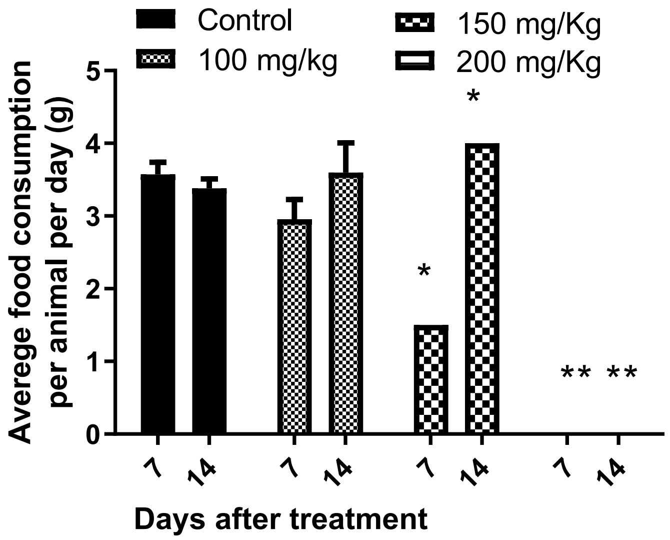

Figure S1. Acute toxicity study shows the mean body weight variation (Fig. S1A) and food consumption (Fig. S1B). Three groups of 3 mice were treated intraperitoneally with a dose (100 mg/Kg, 150 mg/Kg; 200 mg/Kg) of substance **6a** and followed for 14 days to analyze morbidity and mortality, euthanasia, necropsy and histology were performed. A: Average food consumption during treatment, the error bar corresponds to the standard deviation. B: Mean body weight of each group during treatment, the error bar corresponds to the standard deviation. *of the 4 animals for 150 mg/kg only 2 survived after the 7th day. **The 3 animals treated with 200 mg/kg died before the 7th day.

Cell cycle Analysis

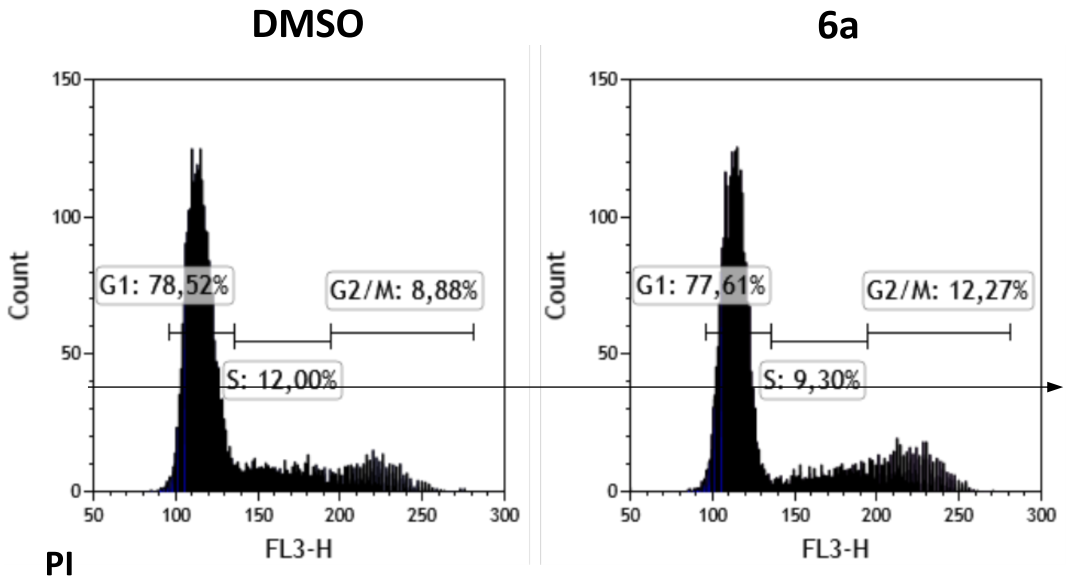

Figure S2. Differences in cell cycle distribution. Cell cycle distribution was analyzed after propidium iodide staining and FACS analyses. SCC9 lineage cells were plated in a 6-well plate (5 x 105 cells/well). The phases (sub G1), G0/G1, S and G2 of the cell cycle were classified based on the DNA content after staining with iodide of propidium (PI). Each image is representative of at least three independent experiments.

Some antitumor compounds can bind to DNA with the possibility of generating a change in the dynamics of the cell cycle, this arrest in some phases of the cycle has already been evidenced in some naphthoquinones. For example, naphthoquinone doxorubicin is capable of inducing a cell cycle arrest in the G0/G1 or G2 phases [13]. The naphthoquinone plumbagin, capable of inducing cell death by autophagy, acts in the cell cycle with an arrest in the G2/M phase [14]. In this way, the effect the antiproliferative effect of compound **6a** was also evaluated by analyzing the cell cycle in flow cytometry (Fig. S2). The observed results show that there was no significant difference in the number of cells in the G1, S and G2/M phases after 48 hours of treatment, the same was observed for the DMSO control. This indicates that the treatment with compound **6a** does not interfere with the cell cycle.

References

1. Trott, O.; Olson, A.J. AutoDock Vina: Improving the speed and accuracy of docking with a new scoring function, efficient optimization, and multithreading. *J. Comput. Chem.* **2009**, *31*, 455-461.

2. Costa, D.C.S.; de Almeida, G.S.; Rabelo, V.W.-H.; Cabral, L.M.; Sathler, P.C.; Abreu, P.A.; Ferreira, V.F.; da Silva, L.C.R.P.; da Silva, F.C. Synthesis and evaluation

of the cytotoxic activity of Furanaphthoquinones tethered to 1H-1,2,3-triazoles in Caco-2, Calu-3, MDA-MB231 cells. *Eur. J. Med. Chem.* **2018**, *156*, 524-533.

3. Chipoline, I.C.; da Fonseca, A.C.C.; da Costa, G.R.M.; de Souza, M.P.; Rabelo, V.W.-H.; de Queiroz, L.N.; de Souza, T.L.F.; de Almeida, E.C.P.; Abreu, P.A.; Pontes, B.; Ferreira, V.F.; da Silva, F.C.; Robbs, B.K. Molecular mechanism of action of new 1,4-naphthoquinones tethered to 1,2,3-1H-triazoles with cytotoxic and selective effect against oral squamous cell carcinoma. *Bioorg. Chem.* **2020**, *101*, 103984.

4. Babu, M.S.; Mahanta, S.; Lakhter, A.J.; Hato, T.; Paul, S.; Naidu, S.R. Lapachol inhibits glycolysis in cancer cells by targeting pyruvate kinase M2. *PLoS One* **2018**, *13*, e0191419.

5. da Silva, M.N.; Ferreira, V.F.; de Souza, M.C.B.V. Um panorama atual da química e da farmacologia de naftoquinonas, com ênfase na beta-lapachona e derivados. *Química Nova* **2003**, *26*, 407-416.

6. Ferreira, S.B.; Gonzaga, D.T.G.; Santos, W.C.; Araújo, K.G.L.; Ferreira, V.F. ß-Lapachone: Medicinal chemistry significance and structural modifications. *Rev. Virtual Quím.* **2010**, *2*, 140-160.

7. Gurbani, D.; Kukshal, V.; Laubenthal, J.; Kumar, A.; Pandey, A.; Tripathi, S.; Arora, A.; Jain, S.K.; Ramachandran, R.; Anderson, D.; Dhawan, A. Mechanism of inhibition of the ATpase domain of human topoisomerase IIα by 1,4-benzoquinone, 1,2-naphthoquinone, 1,4-naphthoquinone, and 9,10-phenanthroquinone. *Toxicol. Sci.* **2012**, *126*, 372-390.

8. Wellington, K.W. Understanding cancer and the anticancer activities of naphthoquinones-a review. *RSC Adv.* **2015**, *5*, 20309-20338.

9. Zu, X.; Xie, X.; Zhang, Y.; Liu, K.; Bode, A.M.; Dong, Z.; Kim, D.J. Lapachol is a novel ribosomal protein S6 kinase 2 inhibitor that suppresses growth and induces intrinsic apoptosis in esophageal squamous cell carcinoma cells. *Phytother. Res.* **2019**, *33*, 2337-2346.

10. Chen, J.; Xie, J.; Jiang, Z.; Wang, B.; Wang, Y.; Hu, X. Shikonin and its analogs inhibit cancer cell glycolysis by targeting tumor pyruvate kinase-M2. *Oncogene* **2011**, *30*, 4297-4306.

11. Zhao, X.; Zhu, Y.; Hu, J.; Jiang, L.; Li, L.; Jia, S.; Zen, K. Shikonin Inhibits Tumor Growth in Mice by Suppressing Pyruvate Kinase M2-mediated Aerobic Glycolysis. *Sci. Rep.* **2018**, *8*, 1-8.

12. Larsen, T.M.; Benning, M.M.; Rayment, I.; Reed, G.H. Structure of the Bis(Mg2+)-ATP-oxalate complex of the rabbit muscle pyruvate kinase at 2.1 Å resolution: ATP binding over a barrel. *Biochemistry* **1998**, *37*, 6247-6255.

13. Lüpertz, R.; Wätjen, W.; Kahl, R.; Chovolou, Y. Dose- and time-dependent effects of doxorubicin on cytotoxicity, cell cycle and apoptotic cell death in human colon cancer cells. *Toxicology* **2010**, *271*, 115-121.

14. Ma, X.; Yin, X.; Liu, H.; Chen, Q.; Feng, Y.; Ma, X.; Liu, W. Antiproliferative activity of plumbagin (5-hydroxy-2-methyl-1,4-naphthoquinone) in human gastric carcinoma cells is facilitated via activation of autophagic pathway, mitochondrialmediated programmed cell death and inhibition of cell migration and invasion. *J. BUON* **2019**, *24*, 2000-2005.
